# Musculoskeletal and physiological responses to vortex wave stimulation in older adults

**DOI:** 10.1113/JP291437

**Published:** 2026-08-24

**Authors:** Alexander Waddell, Marie Korzepa, Archie E. Belfield, Ben Fry, Rickson C Mesquita, David Smith, Jonathan Quinlan, Leigh Breen

**Affiliations:** ^1^ School of Sport, Exercise & Rehabilitation Sciences University of Birmingham Birmingham UK; ^2^ School of Computer Science University of Birmingham Birmingham UK; ^3^ School of Mathematics University of Birmingham Birmingham UK; ^4^ NIHR Birmingham Biomedical Research Centre, University Hospitals Birmingham NHS Foundation Trust University of Birmingham Birmingham UK; ^5^ Diabetes Research Centre, Leicester General Hospital University of Leicester Leicester UK; ^6^ NIHR Leicester Biomedical Research Centre Leicester UK

**Keywords:** human physiology, sarcopenia, skeletal muscle

## Abstract

**Abstract:**

Reductions in musculoskeletal mass and function can occur with ageing and disuse. While resistance exercise training is known to minimise these detriments, it is not always feasible. Vortex wave stimulation (VWS) may mitigate skeletal muscle decline in such scenarios. The aim of the present study was to explore the acute physiological and metabolic effects of VWS in healthy older adults. Fourteen participants consumed deuterium oxide (D_2_O) stable oral isotope tracer for 7 days. Serial skeletal muscle biopsies were obtained to measure integrated rates of myofibrillar (iMyoPS) and sarcoplasmic (iSarcPS) muscle protein synthesis and regulatory signalling over ∼48 h before (habitual) and after two consecutive‐day bouts of VWS. Myoelectrical activity and muscle oxygenation during VWS, and pre‐post changes in peripheral blood flow and concentrations of inflammatory and bone turnover markers were also measured. There was an increase in iMyoPS (0.23% day^−1^, *P* = 0.025), but not iSarcPS (0.07% day^−1^, *P* = 0.582) above habitual rates following VWS. There was no difference in the expression of anabolic signalling proteins, nor peripheral blood flow following VWS. Myoelectrical activity increased during VWS (9.47 µV, *P* = 0.010), while muscle oxygenation decreased at the gastrocnemius (*P* = 0.001) and quadriceps (*P* = 0.017). There was a reduction in P1NP (8.27 µg L^−1^) and lactate (0.84 mmol L^−1^) concentration post‐VWS (both *P* < 0.001), while there was no difference in other biomarkers. This is the first study to demonstrate that VWS elicits acute physiological responses congruent with musculoskeletal adaptive remodelling, which warrants further exploration.

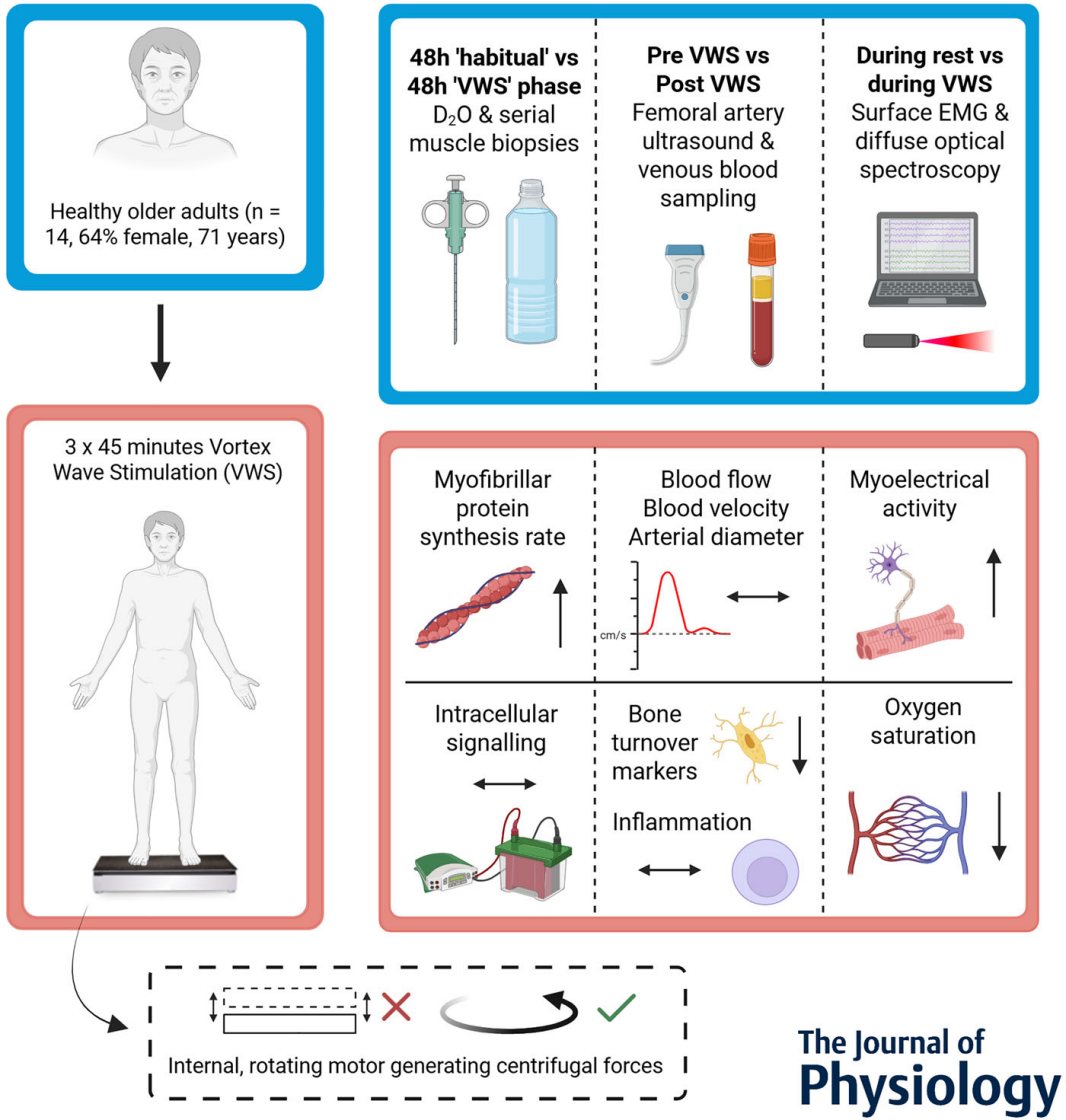

**Key points:**

- Resistance exercise is known to mitigate skeletal muscle atrophy associated with ageing and/or disuse but is not always feasible.
- We investigated, for the first time, the effects of a novel mechanical stimulus – vortex wave stimulation (VWS) – on integrated rates of myofibrillar and sarcoplasmic muscle protein synthesis, alongside other musculoskeletal and physiological outcomes, in healthy older adults.
- We demonstrated that VWS elevated myofibrillar, but not sarcoplasmic muscle protein synthesis compared to baseline, while increasing myoelectrical activity and reducing muscle oxygen saturation, lactate and bone turnover markers.
- These findings suggest VWS elicits acute physiological responses congruent with musculoskeletal adaptive remodelling.

## Graphical abstract

**Abstract figure legend** We explored the acute physiological effects of a novel mechanical stimulus, vortex wave stimulation (VWS), in healthy older adults. Using deuterated water and serial skeletal muscle biopsies, we showed that VWS elevated integrated rates of myofibrillar, but not sarcoplasmic muscle protein synthesis, with no changes observed in protein expression. Vascular ultrasound revealed no changes in femoral blood flow, while venous blood sampling demonstrated a reduction in bone turnover markers. Surface electromyography indicated an elevation in myoelectrical activity during VWS, and diffuse optical spectroscopy was utilised to demonstrate reductions in lower limb muscle oxygen saturation. Created in https://BioRender.com.

## Introduction

The human musculoskeletal system undergoes several age‐related impairments, including reduced muscle mass, strength and function – termed sarcopenia. Bone health is also compromised in ageing, with increased incidence of reduced bone density (osteopenia) and progressive bone loss (osteoporosis) heightening the risk of fracture (Holroyd et al., 2008). It is predicted that by 2050, the number of people over the age of 60 years globally will double (World Health Organization, 2024), which will drive an unprecedented rise in sarcopenia, frailty and bone fracture prevalence, with dire socioeconomic consequences. Indeed, hospitalisation due to sarcopenia is thought to cost the US $40.4 billion annually (Goates et al., 2019), while hip fracture, a common consequence of falls, costs the UK National Health Service approximately £1.25 billion per year (Goh et al., 2026). Furthermore, both muscle weakness and hip fracture hold significant implications for all‐cause mortality, quality of life and non‐communicable disease risk (Damluji et al., 2023).

The maintenance of skeletal muscle mass is predominantly regulated by the homeostatic balance between muscle protein synthesis (MPS) and breakdown (MPB). When rates of MPB consistently exceed that of MPS, muscle mass declines. Thus, anabolic stimuli that elevate MPS, such as mechanical loading or nutrient intake, are vital to achieve protein balance or accretion (Lim et al., 2022). However, many older adults demonstrate a blunted response to such stimuli, termed ‘anabolic resistance’, which may underpin sarcopenia progression (Brook et al., 2016). Bone mass is similarly regulated, depending on a balance between the activity of osteoblasts (which form new bone) and osteoclasts (which break down or resorb old bone). An imbalance between the two will therefore exacerbate the progression of osteoporosis (Xu et al., 2023). Thus, it is important to explore interventions that target these mechanisms in order to mitigate sarcopenia and/or osteoporosis.

Whilst resistance exercise training is known to improve/maintain musculoskeletal health, particularly in older adults and during disuse episodes (Dulac et al., 2025; Smeuninx et al., 2025), it can be impractical in clinical settings where mobility is limited (e.g., during bed rest, intensive care or post‐operative stay) or where there is concern for injury or pain. Further, equipment is typically large and unportable, thus limiting its applicability and accessibility. As such, the development of alternatives that deliver an adaptive stimulus to the musculoskeletal system is essential to achieve widespread societal benefit. Neuromuscular electrical stimulation has demonstrated effectiveness in ameliorating disuse‐induced muscle atrophy; however the effects may be localised to the target muscle (Dirks et al., 2014; Fuchs & van Loon, 2025). Similarly, strategies such as blood flow restriction remain contentious in a disuse context (Fuchs et al., 2025). Further exploration of alternative therapeutic methods that are safe, effective and physiologically beneficial is therefore warranted.

Vortex wave stimulation (VWS) is delivered via a stationary standing platform that generates centrifugal forces exerted at the point of contact. This technology differs from whole body vibration (WBV), where an oscillatory plate, either via alternating left and right or uniform up and down vertical displacements, produces high frequency (15–60 Hz) vibrations, with amplitudes ranging from 1–10 mm (Cardinale & Wakeling, 2005). Conversely, VWS generates centrifugal forces via an internal rotating disc, which produces motions of a similar frequency (15–50 Hz), but much lower amplitudes (0.01–0.2 mm), with the direction of force being continuously rotating (Appendix A). This rotating force vector thus results in a mechanical stimulus distinct from WBV. Due to the low amplitude movements which are not visible to the naked eye, individuals can remain standing on the device (usually in a neutral, comfortable stance) for extended periods of time. Compared to WBV protocols which frequently consist of multiple sets of 1–3 min (Fischer et al., 2019), a session of VWS typically consists of 45–60 min of continuous stimulation. Previous research has demonstrated clear efficacy during stall rest in equine models (akin to human bed rest). Specifically, horses exposed to VWS during 8 weeks of stall rest saw increases in muscle thickness and attenuated loss of bone thickness and area (Greene et al., 2019). Furthermore, VWS has been utilised in private clinics and care homes within the UK, all demonstrating efficacy in vulnerable and frail populations, with self‐assessed reductions in pain/discomfort, increased mobility and improved mental health regularly reported (private clinic unpublished observations). However, there is currently no empirical evidence of the physiological and metabolic response to VWS in humans or understanding of the underlying mechanisms. Elucidating the acute effects of VWS may provide further rationale to explore whether chronic, repeated exposure results in any meaningful effects on the musculoskeletal system.

Herein, we investigated for the first time, the physiological effects of two consecutive‐day bouts of VWS in otherwise healthy older adults. We used serial skeletal muscle biopsies and stable isotope tracer methodology to determine the impact of VWS on integrated rates of myofibrillar and sarcoplasmic muscle protein synthesis (iMyoPS and iSarcPS, respectively). We also assessed relevant canonical intramuscular signalling pathways, systemic markers of bone turnover, inflammation, lower limb blood flow, muscle oxygen saturation and electrical activity. We hypothesised VWS would result in a modest but statistically significant increase in MPS rates, muscle electrical activity and peripheral blood flow.

## Methods

### Participants

Fourteen older adult volunteers agreed to participate in the present study (Table 1). Participants were deemed eligible after completion of a general health questionnaire. Briefly, participants were required to be aged 60–80 years, in good general health, defined as non‐obese (BMI: 19.5–29.9 kg m^−2^), non‐smokers, with no known cardiovascular, metabolic, respiratory or neurological disease. Participants were also required to not be using any medication known to affect protein metabolism, or any anticoagulants or blood thinners. Furthermore, all participants had no prior experience with resistance exercise training. The study was conducted in accordance with the *Declaration of Helsinki*, and ethical approval was obtained from the University of Birmingham (RG_24‐076, ERN_4377) and the Southeast Scotland Research Ethics Committees (24/SS/0086). After confirming eligibility, and being informed of all experimental procedures, all participants provided written informed consent prior to enrolling onto the study. The trial protocol was not prospectively registered.

**Table 1:** Participant baseline characteristics

Characteristic	(*n* = 14)
Age (years)	70.86 ± 5.70
Sex (male/female)	5/9
Height (m)	1.62 ± 0.11
Weight (kg)	66.71 ± 12.44
BMI (kg·m^2^)	25.17 ± 2.75
Body fat (%)	30.73 ± 7.29
Fat‐free mass (kg)	45.81 ± 10.66
Systolic blood pressure (mmHg)	125 ± 9
Diastolic blood pressure (mmHg)	76 ± 6
Total energy intake (kcal·day^−1^)	1759.28 ± 395.56
Protein intake (g·kg^−1^·day^−1^)	0.97 ± 0.27
Carbohydrate intake (g·kg^−1^·day^−1^)	2.87 ± 1.10
Fat intake (g·kg^−1^·day^−1^)	1.07 ± 0.41
Daily steps (*n*)	7544 ± 3494
MVPA (min·week^−1^)	104.17 ± 54.58
Knee extensor MVC (N)	202.49 ± 101.07

Abbreviations: BMI, body mass index; MVC, maximal voluntary contraction; MVPA, moderate to vigorous physical activity.

### Experimental design

**Figure 1:**
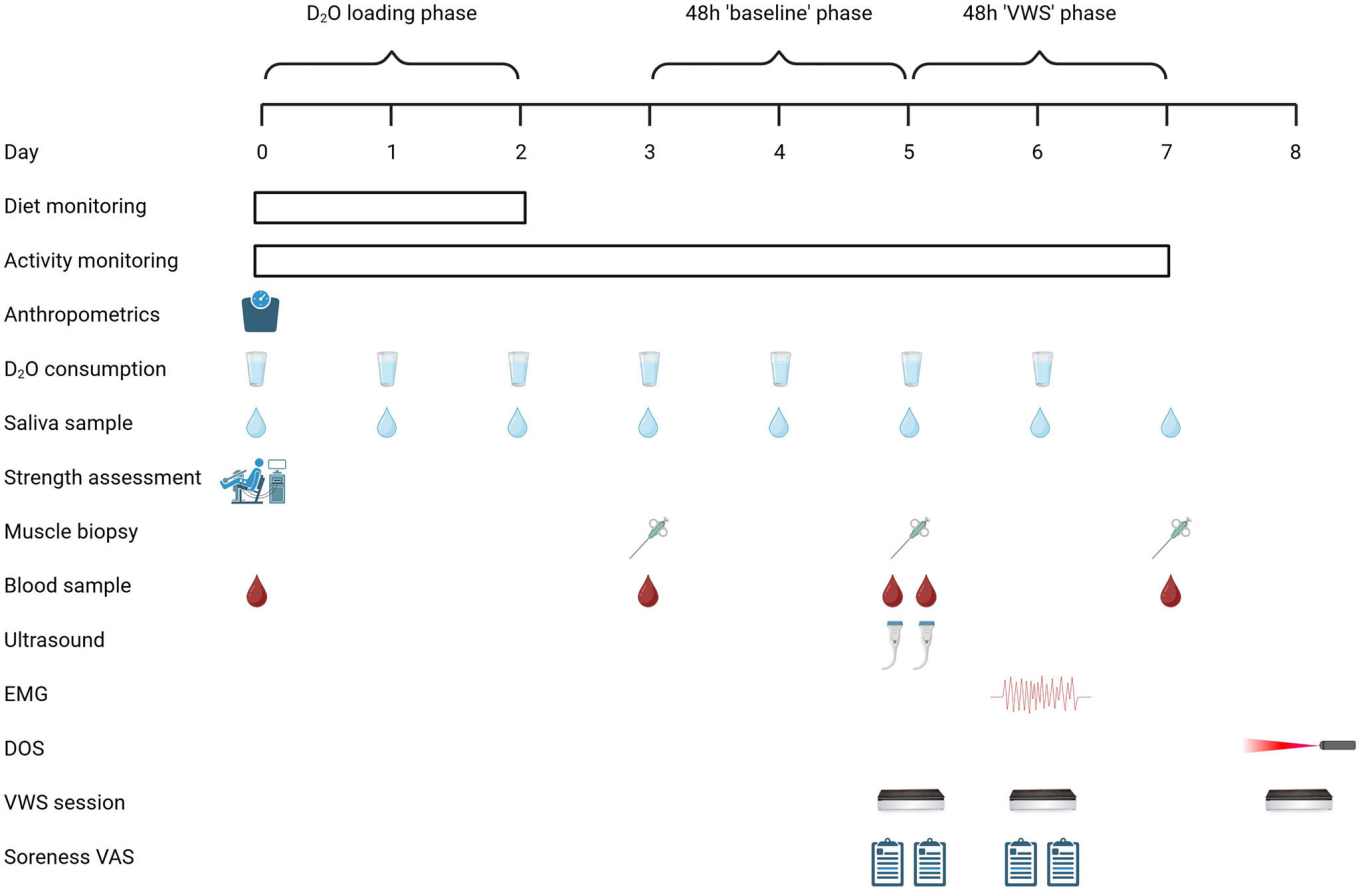
Schematic overview of experimental design D2O, deuterium oxide; VWS, vortex wave stimulation; EMG, electromyography; DOS, diffuse optical spectroscopy; VAS, visual analogue scale. Created in https://BioRender.com

The present study sought to examine the musculoskeletal and physiological responses to two consecutive‐day bouts of VWS. Following screening and informed consent, participants visited the School of Sport, Exercise & Rehabilitation Sciences laboratory on five separate occasions over the course of 8 days. Participants were then subsequently invited back for an additional session of VWS to assess changes in lower limb muscle oxygen saturation (Fig. 1). Participants arrived at a similar time of day for each visit, in an overnight fasted state, having avoided caffeine consumption on the day of testing. Participants were also asked to refrain from alcohol consumption and strenuous exercise for the duration of study involvement, while maintaining their habitual physical activity.

### Experimental protocol

#### Visit 1

Upon arrival to the laboratory, participants underwent an assessment of height, weight and body composition, followed by a resting blood pressure measurement. A baseline saliva sample was then obtained, with further saliva samples being collected daily throughout the study duration. A baseline venous blood sample was then collected, before participants were given a bolus of deuterium oxide (D_2_O) alongside daily top‐up doses (see Experimental procedures for dosing). Participants then completed a maximal voluntary contraction (MVC) of the knee extensors of the dominant leg, as described below. Finally, participants were provided with a wrist‐worn accelerometer to measure physical activity behaviour throughout the study duration (GENEActiv, ActivInsights, Huntingdon, UK), and both a food diary and scale to record dietary habits over the following 3 days.

#### Visit 2

Seventy‐two hours after the previous visit, a second venous blood sample was obtained. Thereafter, a skeletal muscle biopsy was collected from the vastus lateralis (VL) of the non‐dominant leg.

#### Visit 3

Forty‐eight hours after visit 2, another muscle biopsy was obtained, from the VL of the dominant leg. A venous blood sample was then collected, followed by a measurement of blood flow of the superficial femoral artery (SFA) on the dominant leg. Participants then rated their muscle soreness using a visual analogue scale, before completing 45 min of VWS, separated into two bouts of 22.5 min each with a 5‐min comfort break in between. Immediately following the VWS, participants had a repeat measure of SFA blood flow and their muscle soreness. Finally, a second venous blood sample was obtained.

#### Visit 4

Twenty‐four hours after visit 3, participants underwent a second 45‐min bout of VWS while wearing a pair of textile surface electromyography (EMG) shorts (detailed below) to provide an indirect measure of muscle activation.

#### Visit 5

Twenty‐four hours after visit 4, a venous blood sample was obtained. Finally, a second muscle biopsy was collected from the VL of the dominant leg.

#### Visit 6

At a later date (132–283 days following visit 5), participants were invited back to the laboratory for an additional 45‐min session of VWS, during which lower limb muscle oxygen saturation was assessed utilising frequency domain diffuse optical spectroscopy (FD‐DOS) (see below).

### Experimental procedures

#### Body composition

Body composition was determined utilising bioelectrical impedance analysis, providing information on fat mass, body fat percentage and fat‐free mass (BC‐420MA, Tanita Europe, Amsterdam, The Netherlands). Participants were instructed to stand on the scale with bare feet while the measurements were taken.

#### Strength testing

An MVC of the knee extensors was undertaken using an isokinetic dynamometer (Biodex System 3, Shirley, NY, USA) to determine the isometric muscle strength of the quadriceps of the dominant leg. Briefly, participants were seated with the tested limb, chest, and hips stabilised and adjusted so that the axis of rotation of the lever arm was in line with the lateral epicondyle of the femur. All participants completed 3×3 s MVC of the knee extensor, with each contraction separated by a 60 s passive recovery period. Participants were given standardised verbal encouragement for the duration of each 3 s contraction.

#### Vortex wave stimulation

Participants initially underwent two sessions of VWS for the assessments of iMyoPS and iSarcPS, followed by a third session at a later date. Each session was conducted at 90% of the maximal motor input (manufacturer instructions). VWS involves the participant standing on a plate (60 cm × 60 cm × 12 cm), which is supported on the unit by rubber mountings (see Fig. 11). Participants were instructed to remain in a ‘comfortable position’, with a neutral stance and feet shoulder width apart, for 45 min with a short comfort break mid‐way through, with no warmup or cool down required. This was based on the protocol typically utilised within private clinics providing VWS in the UK. The underside of the plate supports a motor and an asymmetrically weighted disc, which rotates at 3000–4000 revolutions per minute, depending on motor input (fixed via potentiometer in the present study) and subject mass. The principle of conversion of linear momentum thereby results in small amplitude gyratory movements of the plate and a continuously rotating centrifugal force being exerted on the participant at the point of contact with the plate, the magnitude of which is approximated by $n\varepsilon \omega^{2}$ where n is the disc mass, ε is the displacement of the centre of mass of the disc, and ω is the rotational frequency in radians per unit time. The resulting force magnitude is in the range of 316–580 N in the present study. See Appendix A for more details.

#### Femoral artery blood flow

Blood flow was measured via high‐resolution duplex ultrasound (Terason U‐smart 3300, Teratech, Burlington, USA) with a 10 MHz linear array probe. Images were obtained following 10 min of supine rest both before and after VWS to ensure standardisation, with probe placement being marked on participants skin following the baseline recording to ensure the same arterial section was measured. Following location of the vessel, longitudinal images of the lumen‐arterial wall were optimised via B‐mode ultrasound. The image was then recorded for 2 min, with simultaneous Doppler velocity assessment collected with the smallest possible insonation angle (always <60°). All recordings were performed by a single trained operator.

Recordings were imported into a specialised custom‐designed edge‐detection wall‐tracking software (Cardiovascular Suite, version 3.4.0, Quipu, Pisa, Italy) to determine arterial diameter and blood flow. Two‐three optimal regions of interest were selected based on image quality. Average values were then calculated for arterial diameter and blood velocity. Blood flow was calculated as the arterial lumen cross‐sectional area × Doppler velocity with the following equation: 3.14 × (diameter/2)^2^ × mean blood velocity × 60. The coefficients of variation between two scans on the same day in our laboratory for arterial diameter, blood velocity and blood flow are 1.44 ± 1.06%, 14.29 ± 9.66% and 14.85 ± 8.57%, respectively.

#### Quadriceps myoelectric activity

Surface EMG was utilised to quantify the electrical potential of the quadriceps and is referred to as ‘myoelectric activity’ henceforth (Vigotsky et al., 2018). Surface EMG was recorded from the VL and vastus medialis (VM) of the right and left quadriceps via textile shorts made of polyamide (71%) and elastane (29%), with bipolar electrodes integrated into the fabric (Mbody, Myontec Ltd, Kuopio, Finland), as previously described (Tikkanen et al., 2013). The electrodes connect wirelessly to a module located on the front of the shorts (MCell, MBMS‐S1.1, Myontec Ltd). Compression sleeves were used to ensure sufficient electrode contact with the skin, and the area underneath the electrodes were sprayed with water to optimise conductivity to maximise signal strength.

The EMG signals were pre‐amplified (1000 Hz) and then band‐pass filtered (2nd order) with cut‐off frequencies of ∼40–300 Hz. EMG data were recorded continuously and digitised synchronously with 12‐bit resolution via an A/D converter. The 1000 Hz signal was first rectified in an automated fashion and then averaged over non‐overlapping 100 ms intervals, similar to the root mean square method. The averaged data was stored in the memory of the MCell at 25 Hz, from which the data was extracted and analysed using customised software (Muscle Monitor, version 3.1.0.4, Myontec Ltd). To obtain average rectified values of EMG amplitude, data from the VL and VM from both legs were first summed. The EMG signal amplitude was then averaged across 1 min of standing on the device while turned off, as well as throughout the VWS session.

#### Frequency domain diffuse optical spectroscopy

Lower limb muscle oxygen saturation, before, during and after a session of VWS was assessed with a commercial FD‐DOS system (Oxiplex, ISS Inc., IL, USA). After calibrating the device on an optical phantom, they were placed on participants calf (gastrocnemius) and thigh (VL) with velcro and muscle tape. Baseline data was then collected for 5 min, with participants standing on the device while turned off. Data was then continuously recorded throughout a 45‐min VWS session, followed by 10 min of recovery following termination of the VWS stimulus.

The device emits near infrared light into the muscle tissue at a known distance from the collector via 16 lasers at two different wavelengths (8 at ∼690 nm and 8 at ∼830 nm). Two photomultiplier tube detectors measure the changes in amplitude (A) and phase shift (θ) of the transmitted light, sampled at ∼1 Hz. The lasers intensity is modulated at 110 MHz and time multiplexed to allow each laser's light to be resolved during detection. Each data collection site used a fibre‐based, commercially available probe connecting 8 sources and 1 detector – allowing data to be collected for each wavelength at 4 source‐detector distances, ρ,(∼2, 2.5, 3, 3.5 cm for the calf and ∼3, 3.5, 4, 4.4 cm for the quadriceps). Sampled amplitude and phase measurements were averaged in 10 s blocks to remove instrumentation noise, then tissue optical absorption was estimated at each sample from the gradients of $\ln(\rho^{2}A)$ *vs*. ρ and θ *vs*. ρ under the assumption of a semi‐infinite homogeneous volume (Fantini et al., 1994). Oxygenated (HbO) and deoxygenated (HbR) haemoglobin concentrations were estimated from the optical absorption values at each wavelength (Durduran et al., 2010) using haemoglobin extinction coefficients taken from Prahl (1999). Total haemoglobin concentration (THC) was calculated as the sum of HbR and HbO, while muscle oxygen saturation ($S_{tO_{2}}$) was calculated as the ratio of HbO to THC. Average values during baseline, the first and last 5 min of VWS (start‐VWS and end‐VWS, respectively), and recovery were then calculated.

#### Isotope tracer protocol

Stable isotopically labelled D_2_O (70 atom %; Cambridge Isotope Laboratories, Inc., Tewksbury, MA, USA) was provided to participants throughout the duration of the study. Deuterium (^2^H) enrichment was measured in muscle tissue and body water (saliva) to calculate habitual (visits 2 and 3) and post‐VWS (visits 3 and 5) fractional synthetic rates (FSR) of muscle protein synthesis via mass spectrometry, as previously described (McKendry et al., 2019). Briefly, to rapidly increase the body water enrichment of ^2^H to ∼0.2% atom per cent excess (APE), participants consumed a bolus dose of D_2_O during visit 1 (2.4 mL kg^−1^ fat‐free mass). Thereafter, to maintain a ‘steady state’ of ∼0.2% APE enrichment of the body water pool throughout the study, participants consumed a daily top‐up dose of D_2_O of 0.3 mL kg^−1^ fat‐free mass. All doses were consumed in the morning, immediately after saliva sample provision. These saliva samples were collected each morning immediately on waking in 10 mL sterile plastic tubes. Samples were temporarily stored in the fridge (∼5°C) and returned to the investigator at the next experimental visit. Saliva samples were subsequently centrifuged at 3011 *g* at 4°C for 10 min to separate salivary debris and pipetted into a 1.5 mL Eppendorf, sealed, and stored at −80°C until later analysis.

#### Skeletal muscle biopsies

Across the study, percutaneous skeletal muscle biopsy samples were obtained (visits 2, 3, and 5) from the VL under local anaesthesia (1% lidocaine) using the Bergström needle technique adapted for suction (Bergström, 1975), as previously reported (Quinlan et al., 2022). Muscle biopsy tissue was quickly rinsed in saline and blotted to remove any visible blood, fat and connective tissue before being immediately snap‐frozen in liquid nitrogen (N_2_) and stored at −80°C until further analysis.

#### Saliva enrichment

Enrichment of ^2^H in the body water pool was analysed via saliva samples and cavity ring‐down spectroscopy. Saliva samples from days 0, 1, 3, 5 and 7 were analysed. The ^2^H enrichment was converted from δ^2^H to Atom% using the following equation:

$$\mathrm{Atom}\%=\frac{100\;\mathrm{AR}\;\times \;\left(\delta^{2}H\;\times \;0.001+1\right)}{1+\mathrm{AR}\;\times \;\left(\delta^{\;2}H\;\times \;0.001+1\right)},$$

where AR represents the absolute ratio constant for ^2^H based on the Vienna standard mean ocean water (isotopic standard value 0.0001559). Atom% values were then converted to APE by correcting for background ^2^H enrichment in baseline saliva samples (day 0).

#### Myofibrillar and sarcoplasmic‐bound alanine determination

Amino acids from both the myofibrillar and sarcoplasmic protein fractions were isolated. Snap‐frozen skeletal muscle samples were weighed (Adventurer AX223, Ohaus Corporation, NJ, USA) and kept in Eppendorfs on liquid N_2_. Muscle tissue was rapidly homogenised in 10 µL of ice‐cold homogenisation buffer (50 mM Tris‐HCL, 1 mM EDTA, 1 mM EGTA, 10 mM B‐glycerophasphate, 50 mM NaF) per mg of tissue. Samples were placed on a shaker (VIBRAX, IKA) at 1000 rpm for 10 min, and subsequently centrifuged at 11,000 *g* for 15 min at 4°C. The resulting supernatant was collected, with 100 µL aspirated into a 1.5 mL Eppendorf, which was combined with 10 µL of an inhibition cocktail (10 mL homogenisation buffer, 75 µL 100 mM sodium orthovanadate, 1× phosphatase inhibitor cocktail (PhosSTOP, Roche) and 1× protease inhibitor (cOmplete mini, Roche)) and stored for both sarcoplasmic protein expression analysis and protein assay to determine total protein content. All remaining supernatant was transferred into a glass tube for iSarcPS analysis. The remaining pellet was collected for the determination of iMyoPS. Thereafter, the pellet was washed to remove residual sarcoplasmic proteins by adding 500 µL ice‐cold homogenisation buffer and centrifuged at 11,000 *g*. After discarding the supernatant, 750 µL 0.3 M NaOH was added and heated to 37°C to solubilise the myofibrillar proteins, before being centrifuged at 18,739 *g* for 10 min at 4°C, with the resulting supernatant collected in glass tubes. Another 750 µL NaOH was added to the pellet and centrifuged at 18,739 *g* for 10 min at 4°C to suspend any residual myofibrillar proteins, which were aspirated into the same glass tube. Both the tubes containing myofibrillar and sarcoplasmic proteins were precipitated with 1 mL 1 M perchloric acid, which were subsequently centrifuged at 3000 *g* for 20 min at 4°C. The supernatant was discarded, and the pellets were cleaned with 2 mL 70% ethanol and centrifuged at 3000 *g* for 10 min at 4°C. To each tube, 1 mL 0.1 M HCL and 1 mL of activated AmberChrom cation‐exchange resin (AG 50WX8, 100–200 mesh hydrogen form, Bio‐Rad, USA) were added, before being closed with screw‐top lids and placed in an oven to hydrolyse at 110°C for 16 h. Samples were poured into separate columns containing activated cation‐exchange resin slurry, by rinsing out with 5 × 1 ml 1 M HCL. To separate out amino acids within the resin, 5 mL double distilled water (DDH_2_O) was added to allow drip through, before amino acids were eluted with 2 × 6 ml 2 M NH_4_OH, dripping into new glass tubes. After forcing any residue through the columns, all glass tubes were dried on a heating block at 90°C under constant N_2_ flow. Thereafter, amino acids were reconstituted in HCL and derivatised before being analysed by gas chromatography‐pyrolysis‐isotope ratio mass spectroscopy (Metabolic Solutions, Nashua, NH, USA).

#### Calculations

Rates of iMyoPS and iSarcPS were calculated using the standard precursor‐product method, from the incorporation of ^2^H‐bound alanine into protein, using the enrichment of saliva‐derived body water as the surrogate precursor labelling between biopsies (Wilkinson et al., 2014). FSR was calculated as follows:

$$FSR\;\left(\%\cdot \mathrm{da}y^{-1}\right)=\left[\frac{\Delta E_{\mathrm{ala}}}{E_{\mathrm{precursor}}\;\times \;t}\right]\times 100,$$

where *ΔE*_ala_ represents the difference in muscle‐bound alanine enrichment between biopsies; *E*_precursor_ is mean body water enrichment (multiplied by 3.7 to correct for the mean number of deuterium moieties incorporated per alanine); and *t* represents the time between muscle biopsies, which was first determined in hours to calculate FSR in % hour^−1^, and subsequently multiplied by 24 to determine daily FSR (% day^−1^).

#### Immunoblotting

Protein expression levels were assessed via western blotting performed on the sarcoplasmic fraction of muscle samples from visit 3 (pre‐VWS) and visit 5 (24 h post‐VWS). Sarcoplasmic protein content was first determined with a DC protein assay (BioRad, Hercules, USA). Samples were then adjusted to 1 µg µL^−1^ in 4× Laemmli sample buffer and heated at 95°C for 5 min. Equal amounts of protein (30 µL) were loaded on a 4–20% gradient precast gel (Criterion TGX, BioRad, Hercules, USA) for electrophoresis at 200 V for 1 h. Samples were then transferred to a polyvinylidene difluoride membrane (Amersham, Cytiva, UK) for 1 h at 100 V. Membranes were subsequently blocked in 3% milk and washed in Tris buffered saline with 0.1% Tween (TBST) prior to overnight incubation at 4°C in the following primary antibodies: total mTOR (CST2983, 1:1000 5% bovine serum albumin (BSA) TBST), phosphorylated (p)‐mTOR^Ser2448^ (CST2983, 1:1000 3% BSA TBST), total protein kinase B (Akt; CST9272, 1:1000 5% BSA TBST), p‐Akt^Ser473^ (CST4060, 1:1000 5% BSA TBST), total eukaryotic elongation factor 2 (eEF2; CST2332, 1:1000 5% BSA TBST), p‐eEF2^Thr56^ (CST2331, 1:1000 5% BSA TBST), total ribosomal protein S6 kinase beta‐1 (p70S6K; CST9202, 1:1000 5% BSA TBST), p‐p70S6K^Thr389^ (CST9205, 1:1000 5% BSA TBST), total eukaryotic translation initiation factor 4E‐binding protein 1 (4EBP1; CST9452, 1:1000 5% BSA TBST), p‐4EBP1^Thr37/46^ (CST2855, 1:1000 5% BSA TBST) and total focal adhesion kinase (FAK; Santa Cruz‐1715, 1:250 5% BSA TBST). Membranes were then washed 3 × 5 min in TBST and incubated in horseradish peroxidase‐linked (HRP) antirabbit IgG (CST7074, 1:5000 TBST for total mTOR and p‐mTOR^Ser2448^, 1:10,000 TBST for all remaining targets) for 1 h at room temperature. Samples were then washed 3 × 5 min in TBST before incubation in chemiluminescent HRP substrate (Millipore Corp, Billerica, USA) and imaged via a G:BOX Chemi XR5T imager (Syngene, Cambridge, UK). The optical density of each protein band was quantified with ImageJ software (National Institute of Health, USA). The intensity of each band was first normalised to a gel control, before being corrected to the total amount of protein loaded, determined via Ponceau S staining. Where appropriate, the phosphorylation of each target protein was expressed relative to its corresponding total protein signal. All changes are presented as relative to baseline values.

#### Muscle glycogen analysis

Where sample availability permitted, portions of snap frozen muscle were freeze‐dried for 48 h (Beta 1.8 LSCplus, Christ, Germany). Subsequently, 2–8 mg of dry muscle was hydrolysed in 500 µL 2.0 M HCL and heated for 2 h at 95°C. After cooling at room temperature, samples were neutralised in 500 µL 2.0 M NaOH and centrifuged at 3011 *g* for 1 min at 4°C. The supernatant was then analysed in duplicate for glucose on an automated clinical chemistry analyser (RX Daytona+, Randox, London, UK). A known concentration of bovine liver glycogen was used to confirm the accuracy of the assay (measured concentration was within 5% of the known concentration).

#### Blood biochemistry

Venous blood samples were obtained at visits 1, 2, 3, and 5. Plasma and serum were collected in EDTA and serum separator tubes (BD Vacutainer, USA), respectively, before being centrifuged (3011 *g* for 10 min at 4°C), pipetted into 1.5 mL Eppendorfs and stored at −80°C until further analysis. Once all samples had been collected, the acute effects of VWS on blood biochemistry were assessed by comparing analyte concentrations immediately before (PRE) and 30 min after (POST) the first session of VWS, as well as 24 h following the second VWS session (48 h).

Plasma samples were analysed by Liverpool Clinical Laboratories (Royal Liverpool Hospital) for concentrations of Procollagen Type I N‐terminal Propeptide (P1NP) and beta‐CrossLaps (CTX) using an automated Cobas e Analyzer (Roche Diagnostics, West Sussex, UK). Enzyme‐linked immunosorbent assays (ELISA) (Biotechne, Minneapolis, USA) were carried out in duplicate to determine plasma concentrations of: C reactive protein (CRP) (DCRP00B), TNF‐α (HSTA00E) and IL‐6 (HS600C). Lactate and glucose concentrations were determined by adding 20 µL of serum to 1 ml haemolysis solution, which was then measured on a Biosen C‐Line analyser (EKF‐diagnostic GmbH, Germany).

#### Diet and physical activity monitoring

Participants were instructed to wear the accelerometers on their non‐dominant wrist for the duration of the study, only removing them when washing. Accelerometers were initialised to sample at a frequency of 10 Hz. Data were extracted using GENEActiv software (V 4.0.8, ActivInsights Ltd, Huntingdon, UK) and analysed using the GENEActiv R markdown tool (version 1.0.4). Non‐wear times, daily step count and physical activity patterns were calculated. Time spent in sedentary, light, moderate and vigorous activity was calculated based on previously published cut‐off values (Esliger et al., 2011). Finally, researchers assessed participants’ habitual food intake. This was determined from a standardised food diary, where participants weighed all food items across 3 days, to obtain a snapshot of food intake representative of habitual eating patterns. Entries were assessed to calculate total energy intake and macronutrient consumption using MyFitnessPal (MyFitnessPal Inc, San Francisco, USA). Participants were instructed to maintain habitual eating patterns throughout the study duration.

### Statistical analysis

The sample size was based on previous findings of resistance exercise‐induced changes in iMyoPS in untrained older adults (1.47 ± 0.064 *vs*. 1.60 ± 0.083% day^−1^) (McKendry et al., 2019). Using G*Power (version 3.1.9.6), 12 participants would provide >99% power to detect this change with a two‐tailed test and α‐level of 0.05. To account for potential attrition or missing data, we aimed to recruit 14 participants.

Data analyses were carried out on GraphPad Prism (V10.5, GraphPad Software, LLC, USA) and R (version 4.4.1) (R Core Team, 2024). Normal distribution was determined by visually inspecting QQ plots, and via the Shapiro‐Wilk test. Two‐tailed, paired samples *t* tests were carried out to assess differences in: habitual and post‐VWS rates of iMyoPS and iSarcPS, pre‐ and post‐VWS protein expression and glycogen content, pre‐ and during VWS myoelectric activity, and immediately pre‐ and post‐VWS blood flow, arterial diameter and blood velocity. Changes in saliva ^2^H, concentrations of circulating blood biomarkers and muscle oxygen saturation were analysed via a one‐way repeated measures ANOVA with time as the within‐subjects factor. Where significant main effects were reported, *post hoc t* tests with Tukey's correction for multiple comparisons were undertaken. Where sphericity was violated, a Greenhouse‐Geisser correction was used. Potential statistical outliers were assessed using Grubb's test. Observations identified as statistically significant outliers were excluded in a sensitivity analysis. A Pearson's correlation coefficient was carried out on change scores for iMyoPS and iSarcPS. Effect sizes for ANOVAs are reported using partial eta squared (η^2^p), while *t* test effect sizes are reported via Cohen's *d*, with 0.2, 0.5 and 0.8 reflecting small, medium and large effects, respectively. Statistical significance was set *a priori* as *P* ≤ 0.05. All values are presented as mean ± SD unless otherwise stated.

## Results

All 14 participants completed the first and second sessions of VWS. Of the 14 participants, one reported an instance of feeling lightheaded during a VWS session, which was terminated prematurely (but still included in the final analysis). No other adverse events were reported for the remaining participants. All but one of the participants reported no increases in muscle soreness pre‐to‐post VWS for the first (visit 3) and second (visit 4) sessions. Physical activity remained consistent throughout the assessment period, with average step counts across the habitual (visits 2–3) and VWS (visits 3–5) phases of 8111.85 ± 4462.41 and 9566.81 ± 4154.26 steps day^−1^, respectively. Eleven participants agreed to return for the third VWS session to determine lower limb muscle oxygen saturation. Skeletal muscle biopsies could not be obtained for two participants, and so *n* = 12 are included for muscle biopsy‐related outcomes. Likewise, complete venous blood sampling could not be obtained for three participants, and so *n* = 11 are included for blood‐related outcomes. Participant characteristics are displayed in Table 1.

### Body water enrichment and integrated protein synthesis rates

**Figure 2:**
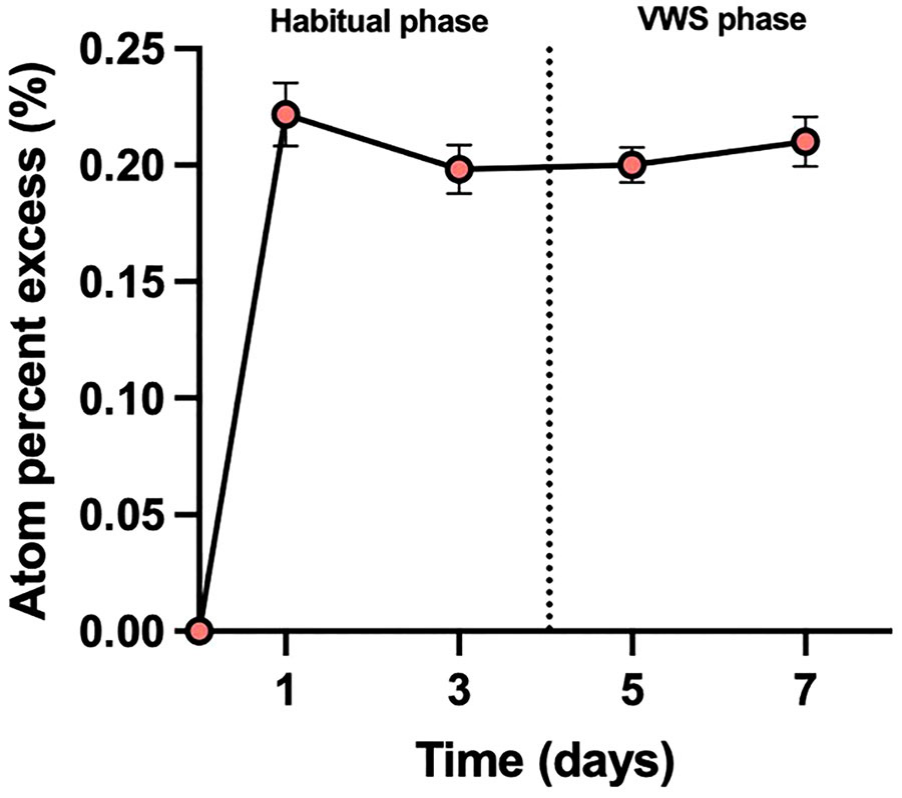
Body water deuterium enrichment throughout the study period Values are presented as mean ± SEM. VWS, vortex wave stimulation.

**Figure 3:**
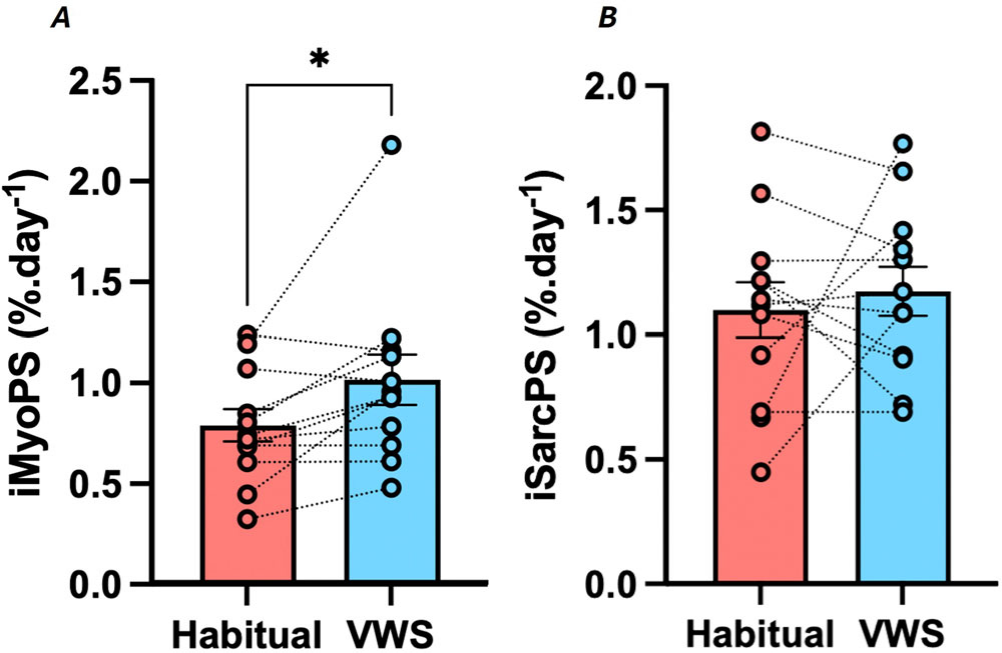
Fractional muscle protein synthesis rates Integrated myofibrillar (*A*) and sarcoplasmic (*B*) protein synthesis rates during 48 h in the habitual state, and after two bouts of VWS. Bars and error bars represent mean ± SEM, and symbols represent individual data points. Data are presented for *n* = 12. ^*^Significant difference (*P *< 0.05). VWS, vortex wave stimulation; iMyoPS; integrated rate of myofibrillar protein synthesis; iSarcPS, integrated rate of sarcoplasmic protein synthesis.

**Figure 4:**
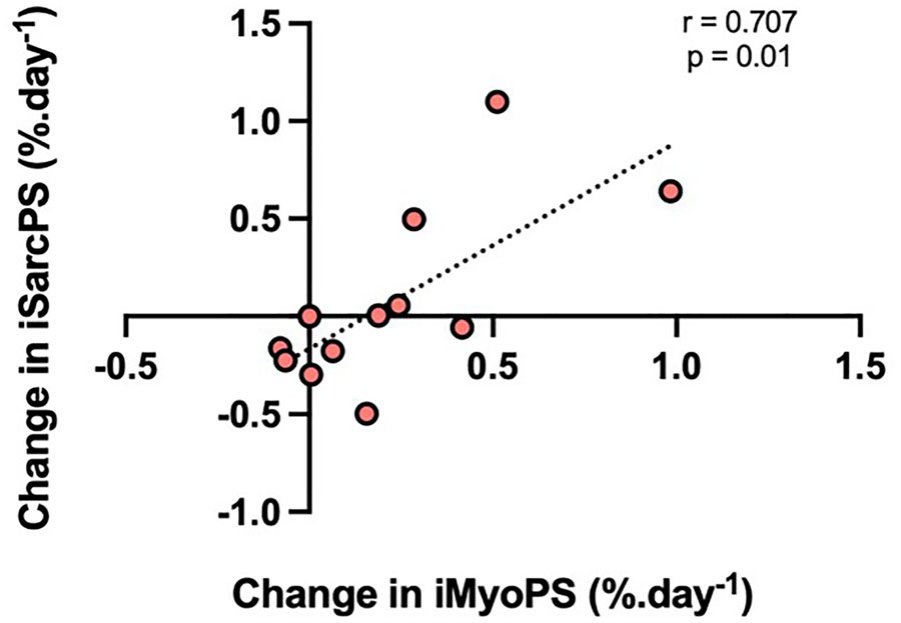
Relationship between change in integrated myofibrillar and sarcoplasmic protein synthesis rates over the post‐VWS period Data were analysed by Pearson correlation coefficient for *n* = 12. iMyoPS; integrated rate of myofibrillar protein synthesis; iSarcPS, integrated rate of sarcoplasmic protein synthesis.

Saliva ^2^H enrichment significantly increased above basal values 24 h after the first dose of D_2_O (*P* < 0.001). Steady state enrichment was maintained throughout the remainder of the study (see Fig. 2). Rates of iMyoPS increased significantly in response to VWS (mean difference (MD) = 0.23% day^−1^, 95% CI: 0.03–0.42, *P* = 0.025, *d* = 0.63). After identifying and removing a high‐responding outlier, statistical significance remained, albeit reduced in magnitude (MD = 0.16% day^−1^, 95% CI: 0.03–0.29, *P* = 0.024, *d* = 0.64). There was no evidence of a difference in rates of iSarcPS following VWS (MD = 0.07% day^−1^, 95% CI: −0.21–0.36, *P* = 0.582) (Fig. 3). Changes in iMyoPS were significantly correlated with changes in iSarcPS (*r* = 0.71, *P* = 0.010, Fig. 4).

### Muscle glycogen concentration

**Figure 5:**
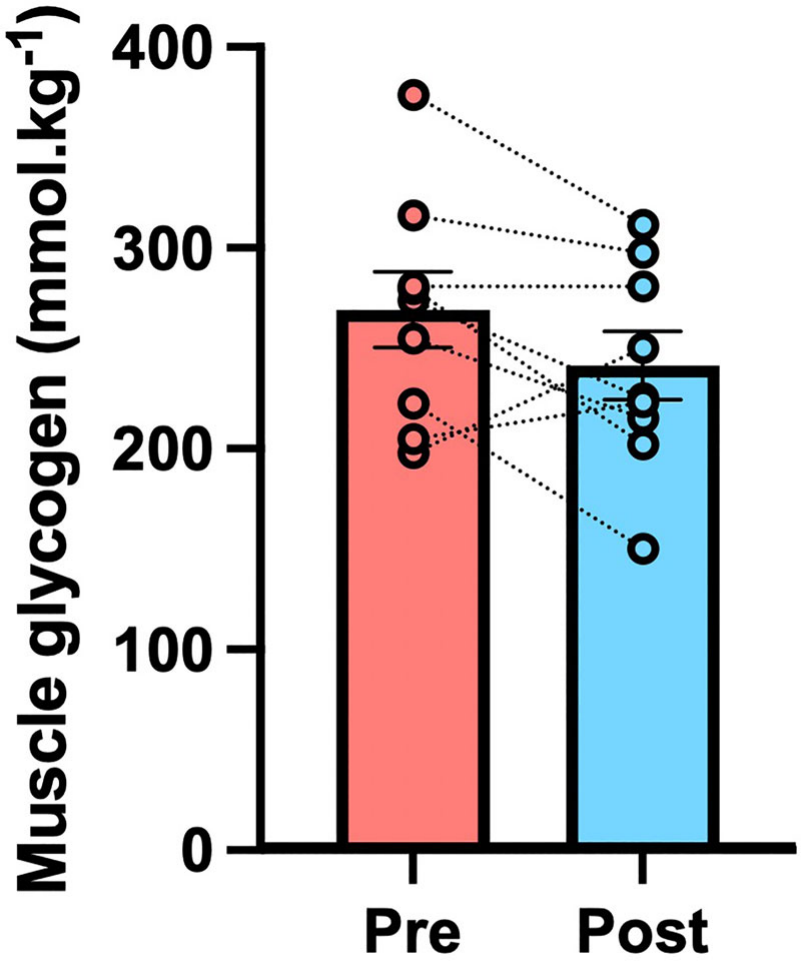
Muscle glycogen concentration pre‐VWS, and following two bouts of VWS (post) Bars and error bars represent mean ± SEM, and symbols represent individual data points. Data are presented for *n* = 9. VWS, vortex wave stimulation.

There was no evidence of a difference in muscle glycogen concentrations between pre‐ and post‐VWS biopsy samples (MD = −27.78 mmol kg^−1^, 95% CI: −61.88–6.33, *P* = 0.097, Fig. 5).

### Intracellular protein expression

**Figure 6:**
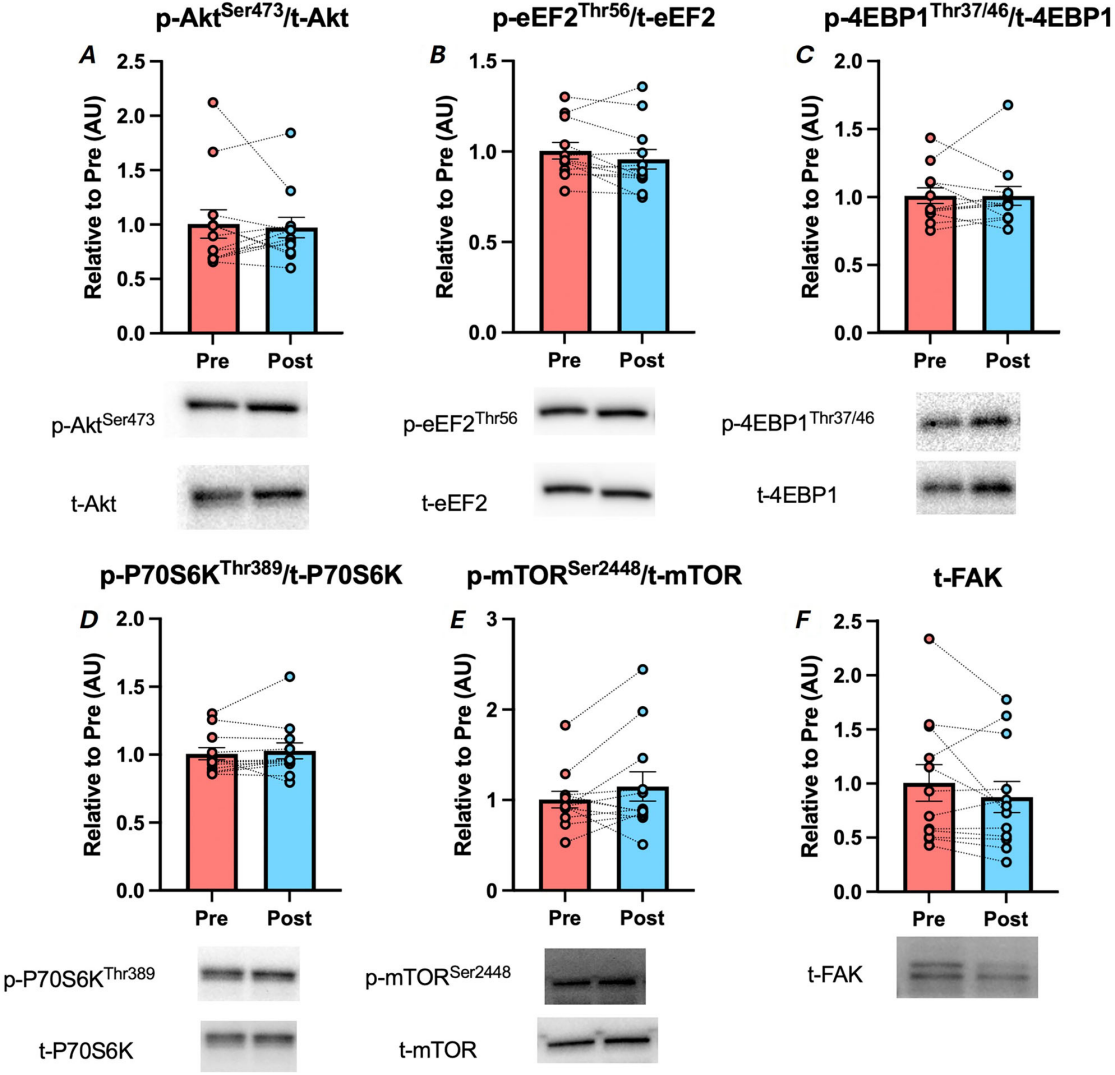
Expression of proteins regulating anabolic signalling Change in protein expression for p‐Akt^Ser473^/t‐Akt (*A*), p‐eEF2^Thr56^/t‐eEF2 (*B*), p‐4EBP1^Thr37/46^/t‐4EBP1 (*C*), p‐P70S6K^Thr386^/t‐P70S6K (*D*), p‐mTOR^Ser2448^/t‐mTOR (*E*) and t‐FAK (*F*) from pre‐VWS to 24 h following the second bout of VWS. Data are expressed as fold change relative to pre‐VWS, where bars and error bars represent mean ± SEM, and symbols represent individual data points. Data are presented for *n* = 12. VWS, vortex wave stimulation.

There was no evidence of a difference in either phosphorylated, total, or phosphorylated/total expression for any proteins analysed within the sarcoplasmic fraction (all *P* > 0.05, Fig. 6).

### Blood biochemistry

Blood biomarker concentration data are shown in Table 2. There was a significant time effect for P1NP (η^2^ *P* = 0.81, *P* < 0.001). *Post hoc* tests revealed that values were significantly reduced post‐VWS (MD = 8.27 µg L^−1^, 95% CI: 5.20–11.35, *P* < 0.001, *d* = 0.40), which returned to pre‐VWS levels at 48 h. There was a significant time effect for CTX (η^2^ *P* = 0.28, *P* = 0.049), however *post hoc* comparisons were not significant. There was no significant time effect for P1NP/CTX (*P* = 0.805). There was also no significant time effect for CRP (*P* = 0.518), TNF‐α (*P* = 0.823) or IL‐6 (*P* = 0.562). There was a significant time effect for lactate (η^2^ *P* = 0.53, *P* = 0.001). *Pos hoc* tests revealed that values were significantly reduced post‐VWS (MD = 0.84 mmol L^−1^, 95% CI: 0.46–1.23, *P* < 0.001, *d* = 1.14), which returned to pre‐VWS levels at 48 h. There was no significant time effect for glucose (*P* = 0.908).

**Table 2:** Systemic concentrations of bone turnover markers, inflammatory cytokines and metabolic markers immediately before (pre), after (post), and at 48 h following VWS (*n* = 11)

	Pre	Post	48 h	*P*
Bone turnover markers				
P1NP (µg·L^−1^)	54.73 ± 21.40	46.45 ± 20.53^***^	56.91 ± 23.22	<0.001
CTX (µg·L^−1^)	0.53 ± 0.32	0.49 ± 0.31	0.57 ± 0.37	0.049
P1NP/CTX (µg·L^−1^)	117.50 ± 35.45	111.90 ± 40.27	113.10 ± 30.34	0.805
Inflammatory markers				
TNF‐α (pg·mL^−1^)	1.45 ± 0.53	1.45 ± 0.53	1.37 ± 0.53	0.823
IL‐6 (pg·mL^−1^)	2.14 ±1.00	1.99 ± 1.03	1.96 ± 0.97	0.562
CRP (mg·L^−1^)	1.68 ± 0.74	1.71 ± 0.75	2.18 ± 2.56	0.518
Other				
Lactate (mmol.L^−1^)	2.36 ± 0.87	1.51 ± 0.58^***^	2.10 ± 1.04	0.001
Glucose (mmol.L^−1^)	5.82 ± 0.91	5.90 ± 0.55	5.84 ± 1.00	0.908

Abbreviations: CRP, C reactive protein; CTX, beta‐CrossLaps; IL‐6, Interleukin 6; P1NP, procollagen type 1 N‐terminal propeptide, TNF‐α, tumour necrosis factor alpha; VWS, vortex wave stimulation.

*** Significant difference (*P* < 0.001) from baseline.

### Myoelectric activity

**Figure 7:**
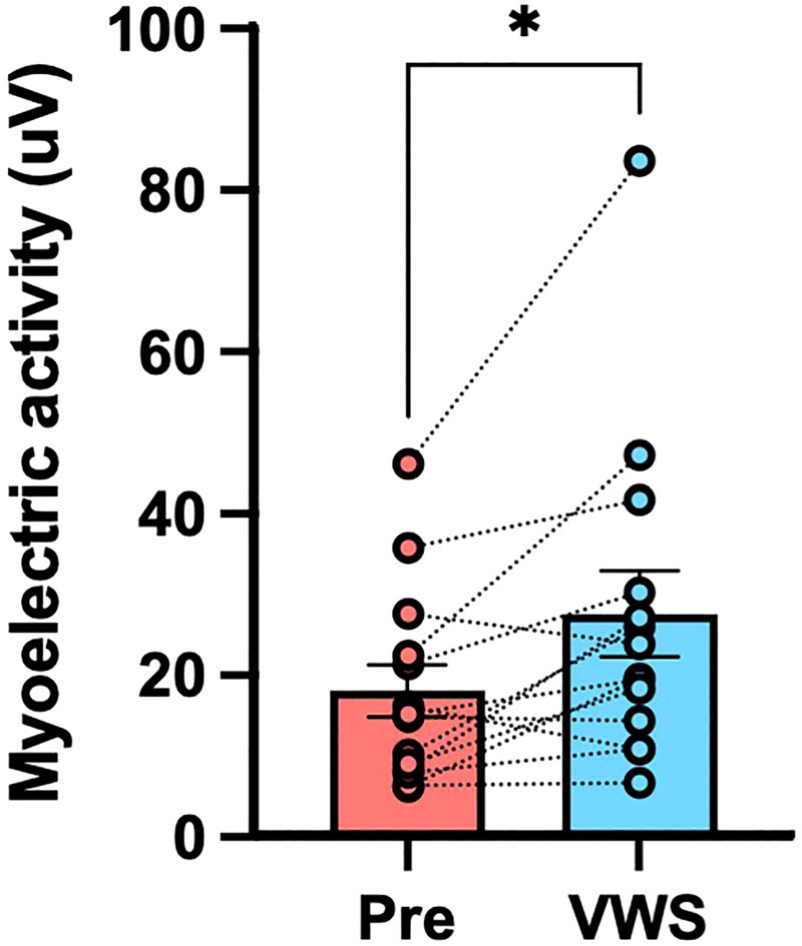
Myoelectric activity immediately prior to (pre) and during a bout of VWS therapy Bars and error bars represent mean ± SEM, and symbols represent individual data points. Data are presented for *n* = 14. ^*^Significant difference (*P < *0.05). VWS, vortex wave stimulation.

Data for myoelectrical activity are presented in Fig. 7. Myoelectric activity was significantly greater during VWS, when compared to rest (MD = 9.47 µV, 95% CI: 2.65–16.30, *P* = 0.010, *d* = 0.58). After identifying and removing a high‐responding outlier, statistical significance remained, albeit reduced in magnitude (MD = 7.32 µV, 95% CI: 1.88–12.70, *P* = 0.013, *d* = 0.69).

### Vascular ultrasound

**Figure 8:**
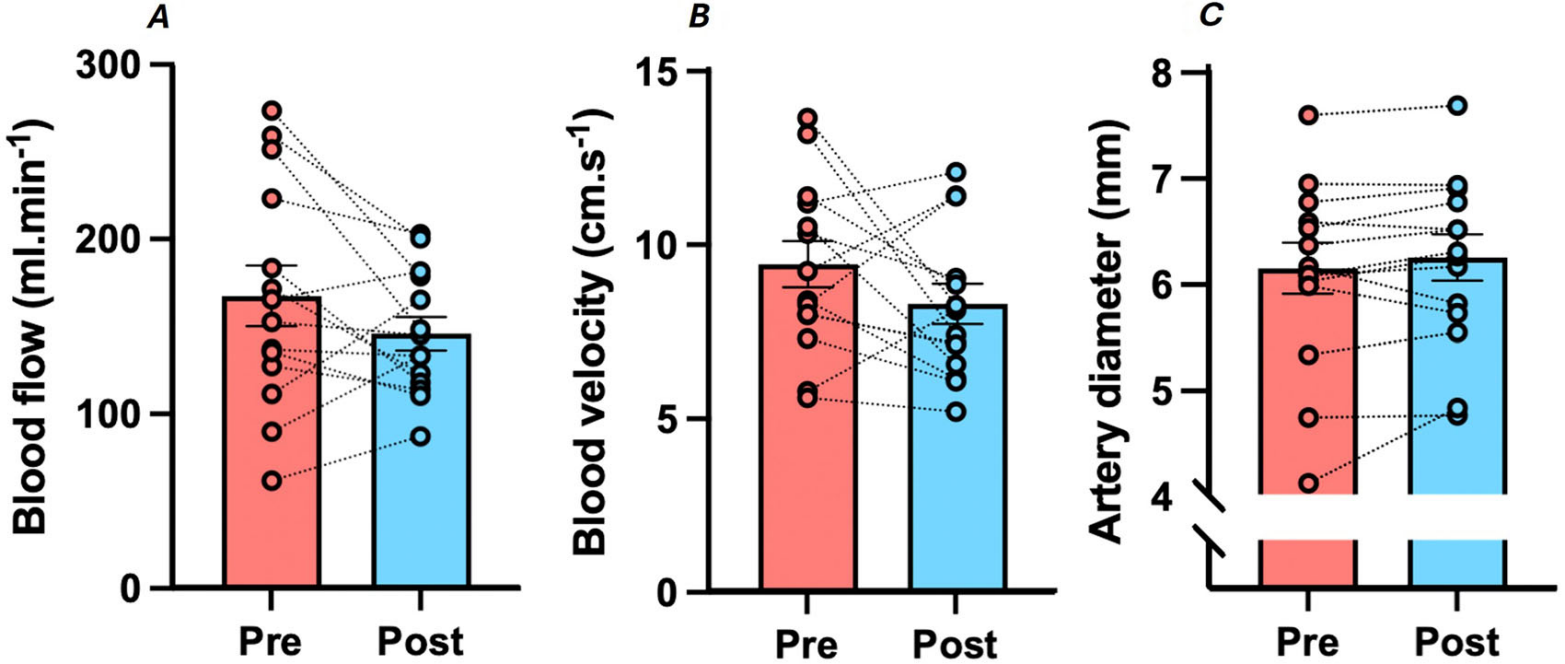
Femoral artery blood flow Superficial arterial blood flow (*A*), blood velocity (*B*) and arterial diameter (*C*) immediately prior to (pre) and following (post) a bout of VWS therapy. Bars and error bars represent mean ± SEM, and symbols represent individual data points. Data are presented for *n* = 14. VWS, vortex wave stimulation.

**Figure 11:**
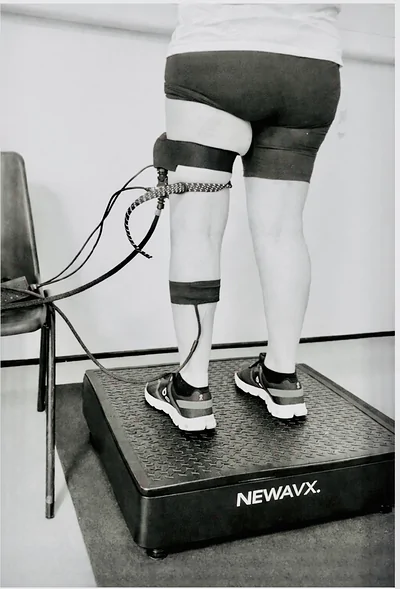
Example of participant undertaking a session of vortex wave stimulation (VWS) This session included simultaneous assessment of muscle oxygen saturation and haemoglobin concentration via frequency domain diffuse optical spectroscopy. Participants remain standing on the device throughout, with readings taken at the quadriceps and gastrocnemius while the device is turned off (baseline), during VWS, and with the device turned off again following VWS (recovery).

Data for ultrasound outcomes are presented in Fig. 8. There was no evidence of a difference in blood flow between pre‐ and post‐VWS time points (MD = −21.63 mL min^−1^, 95% CI: −49.56–6.30, *P* = 0.118). There was also no evidence of a difference in either the diameter of the SFA between time points (MD = 0.10 mm, 95% CI: −0.04–0.246, *P* = 0.158), or in blood velocity (MD = −1.14 cm s^−1^, 95% CI: −2.71–0.44, *P* = 0.143).

### Lower limb oxygen saturation

Data for oxygen saturation and haemoglobin concentrations are shown in Table 3. There was a significant time effect for calf $S_{tO_{2}}$ (η^2^ *P* = 0.43, *P* = 0.001). *Post hoc* tests revealed that values were reduced from baseline at end‐VWS (MD = −2.03%, 95% CI = −0.36 to −3.70, *P* = 0.018, *d* = 0.59) and during recovery (MD = −1.65%, 95% CI = −0.38 to −2.93, *P* = 0.012, *d* = 0.46). There was a significant time effect of calf HbR (η^2^ *P* = 0.48, *P* < 0.001). *Post hoc* tests revealed values at end‐VWS were greater than baseline (MD = 1.16 µM, 95% CI = 0.244–2.08, *P* = 0.014, *d* = 0.19) and start‐VWS (MD = 0.83 µM, 95% CI = 0.20–1.47, *P* = 0.011, *d* = 0.14), and values during recovery were greater than baseline (MD = 0.86 µM, 95% CI = 0.06–1.65, *P* = 0.034, *d* = 0.14). There was no significant time effect for either calf HbO (*P* = 0.111) or calf THC (*P* = 0.253).

**Table 3:** Lower limb oxygen saturation and haemoglobin concentrations at baseline, during the first 5 min of VWS (start VWS), during the last 5 min of VWS (end VWS), and during recovery. (*n* = 11)

	Baseline	Start VWS	End VWS	Recovery	*P*
Calf					
$S_{tO_{2}}$ (%)	62.13 ± 3.89^a,b^	61.36 ± 3.74	60.10 ± 2.98^a^	60.47 ± 3.27^b^	0.001
HbO (µM)	30.18 ± 7.25	29.69 ± 7.18	29.60 ± 6.88	29.39 ± 6.52	0.111
HbR (µM)	18.72 ± 6.12^a,b^	19.04 ± 6.29^c^	19.88 ± 5.89^a,c^	19.57 ± 6.09^b^	< 0.001
THC (µM)	48.89 ± 12.95	48.73 ± 13.10	49.47 ± 12.46	48.96 ± 12.33	0.253
Quadriceps					
StO_2_ (%)	62.18 ± 8.32^a^	61.73 ± 8.34	59.64 ± 8.93^a^	59.62 ± 8.83	0.017
HbO (µM)	34.16 ± 12.74	33.29 ± 12.13	32.95 ± 11.64	33.19 ± 11.40	0.245
HbR (µM)	23.42 ± 16.55^a^	23.46 ± 17.13^b,c^	25.17 ± 17.56^b^	25.35 ± 17.30^a,c^	0.004
THC (µM)	57.58 ± 28.58	56.74 ± 28.59	58.12 ± 28.27	58.54 ± 27.80	0.064

Within a row, values with superscripts of the same letter represent a significant difference (*P* < 0.05).

Abbreviations: HbO, oxygenated haemoglobin; HbR, deoxygenated haemoglobin; $S_{tO_{2}}$, muscle oxygen saturation; THC, total haemoglobin concentration; VWS, vortex wave stimulation.

There was a significant time effect for quadriceps $S_{tO_{2}}$ (η^2^ *P* = 0.41, *P* = 0.017). *Post hoc* tests revealed that values were reduced from baseline at end‐VWS (MD = −2.53%, 95% CI = −0.03–−5.05, *P* = 0.048, *d* = 0.29). There was a significant time effect of quadriceps HbR (η^2^ *P* = 0.45, *P* = 0.004). *Post hoc* tests revealed values at end‐VWS were greater than start‐VWS (MD = 1.72 µM, 95% CI = 0.29–3.14, *P* = 0.019, *d* = 0.10), and values during recovery were greater than baseline (MD = 1.93 µM, 95% CI = 0.20–3.65, *P* = 0.028, *d* = 0.11) and start‐VWS (MD = 1.90 µM, 95% CI = 0.48–3.31, *P* = 0.010, *d* = 0.11). There was no significant time effect for either quadriceps HbO (*P* = 0.245) or THC (*P* = 0.064).

## Discussion

The present study is the first to explore the physiological effects of VWS in human participants. We demonstrate that two consecutive‐day bouts of VWS elevate integrated rates of myofibrillar, but not sarcoplasmic, protein synthesis compared to baseline. We also show that VWS elevates myoelectrical activity, reduces circulating lactate, markers of bone turnover and lowers limb oxygen saturation, with no changes observed in intramuscular anabolic signalling, lower limb blood flow or markers of systemic inflammation. Taken together, these findings suggest VWS represents a novel mechanical stimulus that may induce physiological responses congruent with musculoskeletal adaptive remodelling.

### Muscle related outcomes

Speculation on the mechanisms by which VWS elevates rates of myofibrillar protein synthesis are challenging, as to the best of our knowledge there have been no investigations into the acute molecular responses of skeletal muscle to associated interventions, such as continuous WBV. Whilst providing a different stimulus, the passive nature of WBV makes it a suitable comparison to VWS when discerning potential effects on the musculoskeletal system. The literature surrounding the effects of WBV primarily demonstrate acute increases in neuromuscular activation, which are associated with chronic improvements to physical performance (Tian et al., 2025; Wu et al., 2020). Indeed, we observed elevations to myoelectrical activity during VWS, which based on our previous work, is of similar magnitude to stair descending (Marshall et al., 2020). Thus, it is tempting to suggest VWS may bring about elevations in neuromuscular activation, recruiting muscle fibres necessary to drive the 29% increase in iMyoPS observed herein. However, we did not observe a correlation between the changes in myoelectrical activity and iMyoPS, which may suggest the EMG amplitude is not indicative of neuromuscular activation in this case. Indeed, Morton et al (2019) demonstrate that surface EMG signal during resistance exercise is not related to either type I or II muscle fibre glycogen depletion, nor anabolic signalling protein phosphorylation, suggesting it is not an accurate surrogate for muscle fibre activation. As muscle fibre glycogen depletion is a more established indication of motor unit recruitment (Morton et al., 2019), it could provide more information as to whether VWS elicits any elevations in neuromuscular activation. While we did not observe a statistically significant reduction in muscle glycogen, nor a correlation with change in iMyoPS, there was a consistent pattern of lower glycogen content post‐VWS in most participants. As the post‐VWS biopsy was obtained after 24 h, a more dramatic reduction may have occurred in the immediate hours following VWS, which was then partially restored by the time of the final biopsy (Costill et al., 1981; Namma‐Motonaga et al., 2022). More frequent muscle sampling would help to further characterise the acute glycogen response to VWS.

To further explore potential mechanisms underpinning the muscle protein synthetic response to VWS, we examined changes in the expression of key anabolic signalling proteins involved in the regulation of FSR. However, we did not observe any differences in either phosphorylated or total protein expression following VWS. While some animal models have demonstrated robust elevations in the expression of proteins in the mTOR signalling cascade following WBV (Ceccarelli et al., 2014; Li et al., 2022), this was following multiple days of stimulation. In the present study, we examined protein expression before, and 48 h following VWS, based on the biopsy sampling utilised to assess FSR. Therefore, we may have potentially missed any acute temporal changes in protein expression that did occur following VWS, highlighting the challenges of pairing an integrated free‐living assessment period of FSR, with the comparatively short‐lived intracellular signalling responses that likely precede it. Furthermore, given FSR only increased in the myofibrillar fraction, exploring the expression of individual myofibrillar proteins may provide additional insight into the effects of VWS.

We also demonstrate that pre‐ and post‐VWS sarcoplasmic FSR values were greater than those of the myofibrillar fraction, which is in line with previous findings (Bell et al., 2015; Wilkinson et al., 2014). Interestingly, despite the increase in iMyoPS, iSarcPS was not raised following VWS. Bell et al (2015), who utilised D_2_O to assess integrated rates of protein synthesis, observed iMyoPS to increase following either resistance exercise or high‐intensity interval training (HIIT), whereas iSarcPS only responded to HIIT. Although not directly measured, it was suggested this could be due to the contribution of mitochondrial protein (which resides in the sarcoplasm unless removed during sample processing–see Appendix B), with the metabolic demands of HIIT necessitating an increased mitochondrial FSR. Collectively, it would therefore appear that VWS does not place sufficient demand on the mitochondrial system, although this would need to be confirmed in a future study directly measuring mitochondrial FSR. The higher turnover rate of the sarcoplasmic fraction may also mean the 48‐h assessment period chosen herein may have missed potentially meaningful transient elevations in iSarcPS, as the protein pool is rapidly replenished (Holm et al., 2010). Conversely, the variability observed in the MPS data may indicate a less precise estimate of the effect of VWS, potentially overestimating the magnitude of the iMyoPS response. However, the degree of variability is similar to our previous work (Marshall et al., 2023), and the response was consistent in direction across participants. As such, while the variability warrants a cautious interpretation and replication with larger cohorts, it does not directly imply a false‐positive finding.

### Lower limb blood flow and oxygen saturation

Skeletal muscle oxygenation is based on a balance between the metabolic demand and supply of oxygen to the working muscle (Nyberg & Jones, 2022). A reduction in muscle oxygen saturation therefore represents a mismatch, where either utilisation increases while supply remains the same or declines, or the supply declines while utilisation stays stable. In the present study, we observed reductions in $S_{tO_{2}}$ at both the gastrocnemius (−2.03% from baseline to end‐VWS) and quadriceps (−2.54% from baseline to end‐VWS) during a session of VWS. These changes were accompanied by elevations in HbR (+1.16 µM and +1.75 µM at the gastrocnemius and quadriceps, respectively), and stable THC concentrations, which is consistent with an increase in oxygen extraction within the muscle microvasculature with VWS. While these changes are similar in magnitude and direction to some studies utilising WBV (Games & Sefton, 2013; Okushima et al., 2020), others show no changes in muscle oxygenation, largely depending on the specific vibration protocol delivered (Games et al., 2015). In contrast, the changes in $S_{tO_{2}}$ and HbR in the present study, although following the same direction, were much lower in magnitude than what is typically seen following exercise. Lower limb $S_{tO_{2}}$ is reduced following both knee extension and cycling exercise by ∼15%, while HbR is elevated by ∼40 µM (Okushima et al., 2020). Despite the smaller magnitude of effect in the present study, these findings may indicate an increase in oxygen utilisation and/or local redistribution of blood flow at the microvascular level. While we did not observe any changes in macrovascular blood flow at the femoral artery, this does not necessarily reflect intramuscular blood flow, which has shown to increase during muscle contraction (Izumi et al., 2025; Song et al., 2025). Direct assessment of microvascular blood flow, for example with techniques such as diffuse correlation spectroscopy, would help to resolve these mechanisms further. This combined with an assessment of heart rate would provide greater insight into the acute haemodynamic response during VWS.

### Systemic physiological markers

Bone turnover markers typically respond to acute exercise with an increase in resorption, as mechanical or metabolic stimuli activate osteoclast activity, while bone formation remains unchanged (Dolan et al., 2020). Chronic exercise training then elicits the opposite response, as repeated elevations of bone resorption activate the basic multicellular unit, increasing bone formation (Dolan et al., 2020). The acute response to VWS, with reductions in P1NP and a pattern of lower CTX concentrations is therefore somewhat unexpected. Whilst some evidence suggests postmenopausal women experience a blunted osteogenic effect of exercise (Santos et al., 2017), the reductions in P1NP in the present study occurred consistently across all participants, including both older men and women. Furthermore, with concentrations being reduced following VWS, rather than remaining stable, it is unlikely that the explanation is a blunted response to a potentially osteogenic stimulus. Emerging evidence suggests lactate may act as a signalling molecule mediating bone turnover by promoting osteoblast differentiation (Guo et al., 2025; Zhu et al., 2023). While this raises the possibility that the reduction in lactate concentrations contributed to the concomitant reduction in P1NP, the evidence of a direct relationship between transient fluctuations in lactate and bone turnover markers is limited. Additionally, the similar acute response of CTX concentrations may instead reflect a broader physiological effect of VWS.

Studies examining the acute effects of WBV also demonstrate an initial reduction in markers of both bone formation and resorption. Both Sherk et al (2013) and Bemben et al (2015) observed a reduction in CTX concentrations immediately post‐WBV. *In vitro* evidence suggests that vibration inhibits osteocyte activity, which may explain the reductions in markers of bone resorption (Kulkarni et al., 2013; Lau et al., 2010). However, Harrison et al (2015) also demonstrated a reduction in both P1NP and CTX following WBV in young boys, yet the reductions were also observed in those following a control condition. As both markers of bone formation and resorption are susceptible to diurnal variation (Qvist et al., 2002; Redmond et al., 2016), typically with daytime nadirs, this may have confounded the results and would explain the consistent response given participants were all tested at a similar time of day. Finally, it cannot be discounted that changes in plasma volume may have influenced the results, as we did not assess haemoconcentration in the present study. However, Bemben et al (2015) observed the reductions in CTX following WBV remained when controlling for plasma volume.

We also did not observe any differences in a range of systemic inflammatory biomarkers following VWS, which is consistent with a similar investigation of WBV, showing no changes in plasma CRP immediately following the stimulus (Theodorou et al., 2015). Post‐exercise inflammation can often represent muscle damage, with elevations in cytokines such as IL‐6 observed following high‐intensity exercise (Metsios et al., 2020). Given the lack of increase in inflammatory markers, as well as no consistent muscle soreness following VWS in the present study, it is unlikely that the stimulus induces any discernible muscle damage. However, at certain intensities, exercise can also promote the secretion of cytokines from the muscle, known as myokines, which represent an adaptive response to exercise (Brown et al., 2015; Pedersen et al., 2007). Viewed in this context, the lack of an inflammatory response in the present study suggests that VWS delivers a stimulus that is insufficient to induce myokine secretion. This is further supported by the lack of an increase in serum lactate following VWS. Instead, serum lactate consistently decreased following VWS, which returned to baseline levels by 48 h. As baseline concentrations were slightly above what would typically be expected, this could suggest VWS elicited a response analogous to low intensity active recovery (Menzies et al., 2010), with a concomitant increase in muscle oxygen extraction possibly indicating elevated lactate utilisation. However, in the absence of a time‐matched control, this remains speculative.

### Limitations

Despite the present study being the first to establish some of the potential effects and/or mechanisms of VWS in humans, we acknowledge several limitations. Due to the nature of the study design, with no sham or control condition, we cannot definitively attribute the observed results to the effect of VWS. Furthermore, the muscle biopsy and blood sampling utilised to measure integrative FSR lacks the temporal resolution to reflect a more detailed time course of the physiological response to VWS. However, given the number of outcomes and exploratory nature of the study, we feel more frequent blood and/or biopsy sampling would have placed an unnecessary burden on the participants.

Muscle biopsies were obtained from the VL to assess FSR, enabling direct pairing with EMG and oxygenation measurements at the same anatomical site. However, as participants remained standing during VWS, examining whether responses differ in muscles more directly involved in postural control may provide additional insight. Although we observed comparable muscle deoxygenation in the gastrocnemius, it remains unclear whether other muscle groups, such as those in the core, are similarly or differentially affected by VWS. Our homogenisation protocol omitted protease inhibitors during fractionation to avoid downstream contamination of amino acid quantification in the sarcoplasmic fraction, which could not be washed prior to elution (as it remains a liquid). While all samples were treated identically, some proteolytic degradation may have occurred, potentially affecting the overall protein composition of the samples. Beyond the assessment of muscle soreness, we did not collect other subjective measures such as perceived exertion; inclusion of these, alongside heart rate, could have further contextualised the ‘intensity’ of a VWS session. Although the 3‐day weighed food diary is commonly used to assess habitual intake and participants were instructed to maintain their usual diet, intake was not controlled across the entire study. Consequently, we cannot exclude the possibility that changes in overall energy or protein intake occurred between phases and influenced FSR. Finally, our results are limited to healthy older adults. Those with poorer health or mobility/functional limitation, who arguably are more likely to require passive therapy options, may have experienced different responses. Again, due to the novelty of the present study, with no empirical data on the safety of VWS available, restricting the study to healthy older adults was the most appropriate approach.

### Conclusion

This is the first study to demonstrate some of the acute musculoskeletal and broader physiological effects of a novel mechanical stimulus, VWS, in an older human population. Two consecutive‐day bouts of VWS induced physiological responses congruent with musculoskeletal adaptive remodelling. A comprehensive assessment of the longer‐term physiological adaptation to VWS therapy is warranted to expand on short‐term mechanistic responses presented herein. Specifically, chronic exposure to VWS will clarify whether these acute changes translate to any meaningful effects on muscle mass and/or function in older adults, particularly those undergoing catabolic crises, such as disuse or chronic inflammatory diseases. Such work would help define the potential for VWS to mitigate key musculoskeletal and physiological declines associated with ageing and illness.

## Additional information

## Competing interests

Alexander Waddell, David Smith, Jonathan Quinlan and Leigh Breen have received one or more of the following from NEWAVX Ltd; fees for advisory board work, hospitality, and travel/meeting expenses. Leigh Breen and David Smith hold advisory roles for NEWAVX Ltd, which are non‐salaried. NEWAVX Ltd had no role in the study design, outcome selection, data analysis, or interpretation, but were given the opportunity to comment on the draft manuscript during preparation.

## Author contributions

Conception or design of the work (J.Q., L.B.); Acquisition, analysis or interpretation of data for the work (A.W., M.K., A.E.B., B.F., R.C.M., D.S., J.Q., L.B.); Drafting the work or revising it critically for important intellectual content (A.W., M.K., A.E.B., B.F., R.C.M., D.S., J.Q., L.B.); Approved the final version of the manuscript (A.W., M.K., A.E.B., B.F., R.C.M., D.S., J.Q., L.B.); Agree to be accountable for all aspects of the work (A.W., M.K., A.E.B., B.F., R.C.M., D.S., J.Q., L.B.). All persons designated as authors qualify for authorship, and all those who qualify for authorship are listed.

## Funding

The research was supported by funding from the West Midlands Health Technology Innovation Accelerator to NEWAVX Ltd and Leigh Breen/Jonathan Quinlan.

## Generative AI statement

No generative AI tools were used in the preparation of this manuscript.

## Supporting information


Peer Review History

## Figures and Tables

**Figure 9:**
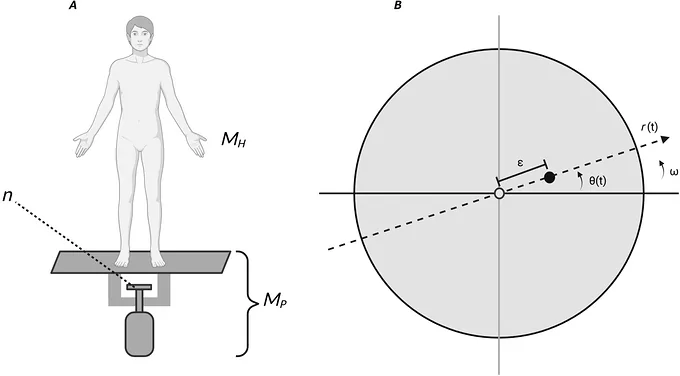
Schematic of the model setup *A*, human participant of mass $M_{H}$, plate/motor assembly of mass $M_{P}$ and disc of mass n. *B*, rotating disc with angular velocity ω centre of mass located off‐centre, displaced by a distance ε in the direction r(t), the latter making an angle θ(t) with a fixed horizontal axis. Created in https://BioRender.com

**Figure 10:**
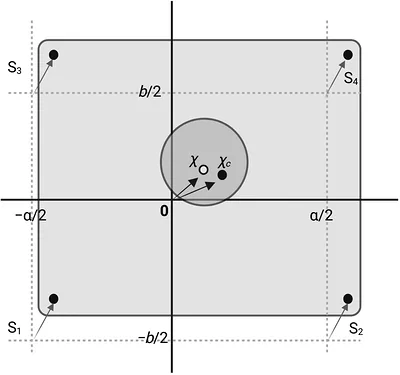
Schematic representation of the geometry and displacement vectors viewed from above The diagram shows the centroid of the plate and disc x, centre of mass of the disc $x_{c}$, displacement vectors of the elastic supports $s_{1},\;\text{…},\;s_{4}$ and support spacings a, b. For visual clarity, displacements are exaggerated. Created in https://BioRender.com

**Figure 12:**
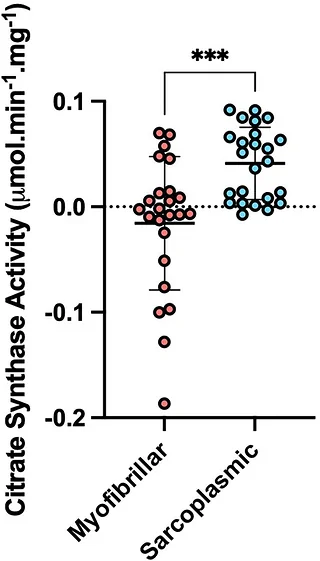
Citrate synthase activity in the myofibrillar and sarcoplasmic fractions of skeletal muscle samples across the whole intervention Lines represent mean ± SD, and symbols represent individual data points. Data are presented for *n* = 24. ^***^Significant difference (*P* < 0.01).

**Figure 13:**
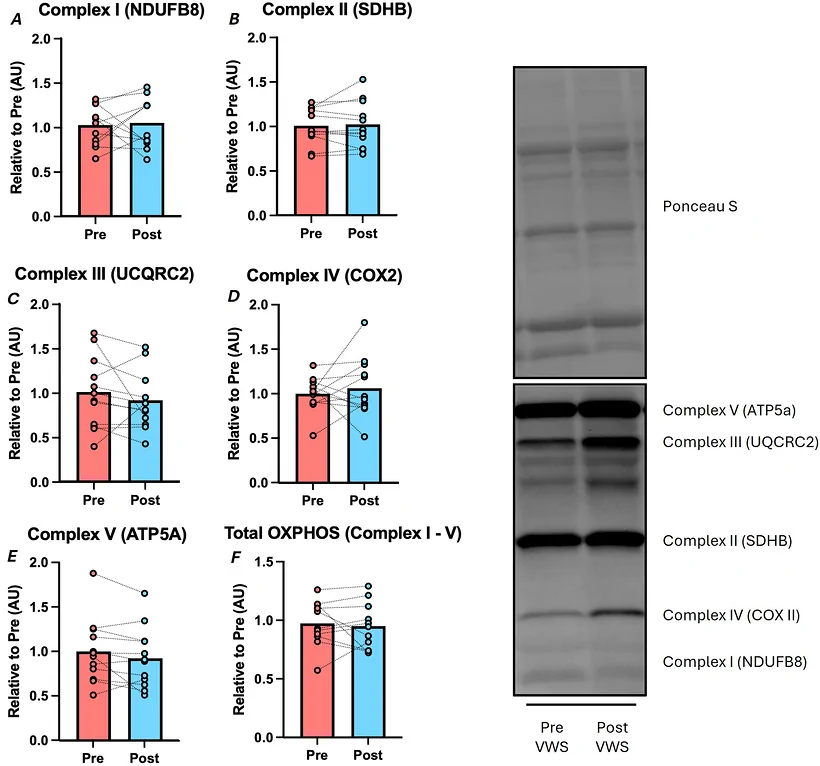
Changes in protein composition Change in protein expression for Complex I (*A*), Complex II (*B*), Complex III (*C*), Complex IV (*D*), Complex V (*E*) and Total OXPHOS (*F*) from pre‐VWS to 24 h following the second bout of VWS. Data are expressed as fold change relative to pre‐VWS, where bars and error bars represent mean ± SEM, and symbols represent individual data points. Data are presented for *n* = 12. VWS, vortex wave stimulation.

## Data Availability

Data are available from the corresponding author upon reasonable request.

## Appendix A

### Model for centrifugal force generation

#### Model set‐up

The mechanical system formed by the device and human participant is modelled as follows:

1. The combination of the plate and attached motor assembly, of mass $M_{P}$ and human participant, of mass $M_{H}$ (Fig. 9*A*). The participant is assumed to rest on the plate in the limit of strong friction and minimal deformation, so that the combined assembly can be treated as a rigid body for the purpose of determining the imposed force.
2. The rotating disc, which has a surface profile ensuring that its mass n is asymmetrically distributed, leading to a displacement of the centre of mass by a distance ε from the axis of rotation (Fig. 9*A*,*B*). The unit direction vector pointing from the disc centroid through the centre of mass is denoted by r(t) where t is time, and the angle between r(t) and a fixed horizontal line is denoted by θ(t) (Fig. 9*B*), so that r(t)=[cosθ(t),sinθ(t),0].
3. The system reaches a limit cycle (steady angular velocity) at which the applied torque balances the resistance torque on the plate, at which the angular radian frequency of the disc is $\dot{\theta }=\omega$ (Fig. 9*B*).
4. Four supports, which are stiff elastic couplings spaced by a distance a along one horizontal axis and b along the other. The couplings are assumed to exhibit linearly elastic behaviour, each with modulus of elasticity k. The positions of the supports relative to an inertial frame are denoted $s_{1}\left(t\right),\;\text{…},\;s_{4}\left(t\right).$ The centroid of the plate is denoted by the vector x and the centroid of the disc by $x_{c}$ (Fig. 10).

#### Linear momentum balance and centrifugal force

Balancing the elastic force exerted by the supports with the rate of change of linear momentum of the full assembly of plate, participant and disc yields the equation:

(A1)
$$-k\sum_{j=1}^{4}s_{j}=n\overset{\cdot \cdot }{x_{c}}+\left(M_{P}+M_{H}\right)\overset{\cdot \cdot }{x}.$$

The left‐hand side of Eqn (A1) can be rewritten as simply −4kx by exploiting rigid body motion of the plate and symmetry in the location of the supports. Applying rigid body mechanics to the right‐hand side, in the limit cycle where $\dot{\theta }=\omega$, the right‐hand side can be expressed as $\left(n+M_{P}+M_{H}\right)\ddot{x}-n\varepsilon \omega^{2}r.$

The resulting equation:

(A2)
$$-4kx=\left(n+M_{P}+M_{H}\right)\;\ddot{x}-n\varepsilon \omega^{2}r,$$

has a periodic particular solution:

(A3)
$$x=\frac{n\varepsilon \omega^{2}}{4k-\left(n+M_{P}+M_{H}\right)\omega^{2}}\;r,$$

which indicates that the motion of the plate can be approximated by small circles of radius $\delta =n\varepsilon {\left[n+M_{P}+M_{H}\right]}^{-1}{\left[1-4k/\left(n+M_{P}+M_{H}\right)\omega^{2}\right]}^{-1},$ which in the high frequency limit ($\omega \gg 2\sqrt{k/(n+M_{P}+M_{H})}$) is approximated simply by $\delta =n\varepsilon {\left[n+M_{P}+M_{H}\right]}^{-1}.$ The direction of the force rotates continuously with the vector r(t).

#### Numerical estimates

Under the motion given by Eqn (A3), the plate and participant are subject to a centrifugal force $F_{c}$ of $\left(M_{P}+M_{H}\right)\ddot{x},$ which has magnitude:

(A4)
$$\left|F_{c}\right|=\frac{n\left(M_{P}+M_{H}\right)\omega^{2}}{n+M_{P}+M_{H}}\;\varepsilon {\left[1-\frac{4k}{\left(n+M_{P}+M_{H}\right)\omega^{2}}\right]}^{-1}.$$

In the high frequency limit and also noting the high mass ratio ($M_{P}+M_{H}\gg n),$ this expression can be approximated by the compact formula:

(A5)
$$\left|F_{c}\right|\approx n\varepsilon \omega^{2}.$$

For the prototype device utilised in the study, n=0.274 kg, $\varepsilon =12\times 10^{-3}$ m and ω≈310−420 rad s^−1^ (3000–4000 rpm), the magnitude of the force is 316–580 N and the amplitude of displacement for a combined participant and plate mass of 90 kg is δ≈0.04mm.

## Appendix B

### Mitochondrial content in myofibrillar and sarcoplasmic muscle fractions

#### Methods

##### Citrate synthase activity assay

Maximal citrate synthase (CS) enzyme activity was determined as a marker of mitochondrial content as previously described (Edwards et al., 2021). A CS reaction buffer [50 mm potassium phosphate (KPI) buffer; pH 7.4, 100 µM DTNB, and 115 µM acetyl‐CoA in DDH_2_O], and spectrophotometer were warmed to 30°C for optimal enzymatic reactions. For baseline measurements, 4 µL of sample and 242 µL of warm reaction buffer were pipetted into a 96‐well microplate, with each sample measured in duplicate. Baseline absorbance was read every 10 s for 2 min at 412 nm in a microplate reader (FLUOstar Omega, BMG Labtech, Aylesbury, UK). Subsequently, 4 µL of oxaloacetate (100 µM final concentration) was added to each well to initiate the reaction before the plate was returned to the spectrophotometer and read again every 10 s for 2 min at 412 nm, to measure the rate of thionitrobenzoate anion (TNB) appearance. Enzyme activity was calculated as: the Δ absorbance/min × 1000/[(ε × volume of sample used in mL) × (sample protein concentration in mg·mL^−1^)], where ε is the extinction coefficient of TNB (13.6). The average enzyme activity of each duplicate was taken forward for analysis. This protocol has been previously validated (Janssen & Boyle, 2019).

##### Immunoblotting

In addition to the protein expression analysis presented in the main manuscript, utilising the same methods we also assessed mitochondrial protein content by incubating membranes overnight at 4°C in total OXPHOS human antibody cocktail (Abcam, cat. no. 110411, 1:1000 TBST). Following overnight incubation, membranes were then washed 3 × 5 min in TBST and incubated in horseradish peroxidase‐linked (HRP) antimouse IgG (Abcam, cat. no.CST7076, 1:10,000 TBST) for 1 hour at room temperature. Samples were then washed 3 × 5 min in TBST before incubation in chemiluminescent HRP substrate (Millipore Corp, Billerica, USA) and imaged via a G:BOX Chemi XR5T imager (Syngene, Cambridge, UK), as described in the main text.

#### Results

##### CS activity assay

Citrate synthase activity was detectable in the sarcoplasmic fraction (0.041 µmol min^−^^1^ mg^−^^1^), whereas activity in the myofibrillar fraction was negligible (−0.016 µmol min^−^^1^ mg^−^^1^). CS activity was significantly greater in the sarcoplasmic *vs*. myofibrillar fraction (MD = 0.057 µmol min^−^^1^ mg^−^^1^, 95% CI: 0.027–0.086, *P* = 0.0003, Fig. 12). However, the lack of detectable CS activity in the myofibrillar fraction is likely a result of the fractionation protocol, with solubilisation in NaOH affecting enzyme integrity.

##### Immunoblotting

Western blot data show the presence of mitochondrial proteins within the sarcoplasmic fraction of skeletal muscle samples. Complexes V, III and II were clearly detected, while complexes I and IV were comparatively faint (Fig. 13), consistent with observations from the antibody manufacturer that sample heating can reduce detection of these proteins (Abcam, 2025). There was no evidence of a difference in protein expression for any of the mitochondrial complexes between pre‐ and post‐VWS (all *P* > 0.05). Due to the fractionation protocol, low protein content and sample availability, we were unable to assess the presence of these proteins in the myofibrillar fraction.

Therefore, these findings demonstrate the presence of mitochondrial complex proteins and CS activity in the isolated sarcoplasmic fractions examined in the present study.
