# Supplementary material for: Musculoskeletal and physiological responses to vortex wave stimulation in older adults
Source: J Physiol. 2026 Aug 24;604(17):7264–86. doi: 10.1113/JP291437 (PMC13532979; doi:10.1113/JP291437)
Supplement: Supplementary file 1 [file TJP-604-7264-s001.pdf]

The Journal of Physiology

Musculoskeletal and physiological responses to vortex wave stimulation in older adults

Alexander Waddell, Marie Korzepa, Archie E Belfield, Ben Fry, Rickson C. Mesquita, David John Smith, Jonathan I Quinlan, and Leigh Breen
DOI: 10.1113/JP291437

Corresponding author(s): Leigh Breen (l.breen@leicester.ac.uk)

The referees have opted to remain anonymous.

Review Timeline:

Submission Date:	27-Mar-2026
Editorial Decision:	01-May-2026
Revision Received:	01-Jul-2026
Editorial Decision:	20-Jul-2026
Revision Received:	21-Jul-2026
Accepted:	28-Jul-2026

Senior Editor: Bettina Mittendorfer

Reviewing Editor: Casper Soendenbroe

Transaction Report:

(Note: With the exception of the correction of typographical or spelling errors that could be a source of ambiguity, letters and reports are not edited. Depending on transfer agreements, referee reports obtained elsewhere may or may not be included in this compilation. Referee reports are anonymous unless the Referee chooses to sign their reports.)

Re: JP-RP-2026-291437 "**Musculoskeletal and physiological responses to vortex wave stimulation in older adults**" by Alexander Waddell, Marie Korzepa, Archie E Belfield, Ben Fry, Rickson C. Mesquita, David John Smith, Jonathan I Quinlan, and Leigh Breen

Dear Dr Breen,

Thank you for submitting your manuscript to The Journal of Physiology. It has been assessed by a Reviewing Editor and by 2 expert referees and we are pleased to tell you that it is potentially acceptable for publication following satisfactory major revision.

Please advise your co-authors of this decision as soon as possible.

The referee reports are copied at the end of this email.

Please address all the points raised and incorporate all requested revisions or explain in your Response to Referees why a change has not been made. We hope you will find the comments helpful and that you will be able to return your revised manuscript within 2 months. If your article is NOT for a Special Issue, you may have 9 months to revise. If you require an extension, please contact journal staff: jp@physoc.org. Please note that this letter does not constitute a guarantee for acceptance of your revised manuscript.

Your revised manuscript should be submitted online using the link in your Author Tasks: Link Not Available. This link is accessible via your account as Corresponding Author; it is not available to your co-authors. If this presents a problem, please contact journal staff (jp@physoc.org). Image files from the previous version are retained on the system. Please ensure you replace or remove any files that are being revised.

If you do not wish to submit a revised version of your manuscript, you must inform our journal staff (jp@physoc.org) or reply to this email to request withdrawal. Please note that a manuscript must be formally withdrawn from the peer review process at one journal before it may be submitted to another journal.

TRANSPARENT PEER REVIEW POLICY: To improve the transparency of its peer review process, The Journal of Physiology publishes online as supporting information the peer review history of all articles accepted for publication. Readers will have access to decision letters, including Editors' comments and referee reports, for each version of the manuscript, as well as any author responses to peer review comments. Referees can decide whether or not they wish to be named on the peer review history document.

ABSTRACT FIGURES: Authors are expected to use The Journal's premium BioRender account to create/redraw their Abstract Figures. Information on how to access this account is here: https://physoc.onlinelibrary.wiley.com/journal/14697793/biorender-access.

This will enable Authors to create and download high-resolution figures. If authors have used the free BioRender service, they can use the instructions provided in the link above to download a high-resolution version suitable for publication.

The link provided should only be used for the purposes of this submission. Authors will be charged for figures created on this account if they are not related to this manuscript submission.

LANGUAGE EDITING AND SUPPORT FOR PUBLICATION: If you would like help with English language editing, or other article preparation support, Wiley Editing Services offers expert help, including English Language Editing, as well as translation, manuscript formatting, and figure formatting at www.wileyauthors.com/eeo/preparation. You can also find resources for Preparing Your Article for general guidance about writing and preparing your manuscript at www.wileyauthors.com/eeo/prepresources.

RIGOUR AND REPRODUCIBILITY: We recommend authors consult The Journal's Rigour and Reproducibility requirements to ensure that their manuscript is suitable for publication: https://physoc.onlinelibrary.wiley.com/pb-assets/hub-assets/physoc/documents/TJP-Rigour-and-Reproducibility-Requirements.pdf

REVISION CHECKLIST:

Check that your Methods section conforms to journal policy: https://jp.msubmit.net/cgi-bin/main.plex?form_type=display_requirements#methods.

Check that data presented conforms to the statistics policy: https://jp.msubmit.net/cgi-bin/main.plex?form_type=display_requirements#statistics.

Upload a full Response to Referees file. To create your 'Response to Referees': copy all the reports, including any comments from the Senior and Reviewing Editors, into a Microsoft Word, or similar, file and respond to each point, using font or background colour to distinguish comments and responses and upload as the required file type.

Please upload two versions of your manuscript text: one with all relevant changes highlighted and one clean version with no changes tracked. The manuscript file should include all tables and figure legends, but each figure/graph should be uploaded as separate, high-resolution files.

You may also upload:

- 'Potential Cover Art' for consideration as the issue's cover image
- Appropriate Supporting Information (Video, audio or data set: see https://jp.msubmit.net/cgi-bin/main.plex?form_type=display_requirements#supp).

We look forward to receiving your revised submission.

If you have any queries, please reply to this email and we will be pleased to advise.

Yours sincerely,

Bettina Mittendorfer
Senior Editor
The Journal of Physiology

REQUIRED ITEMS

- Author photo and profile. First or joint first authors are asked to provide a short biography (no more than 100 words for one author or 150 words in total for joint first authors) and a portrait photograph. These should be uploaded and clearly labelled together in a Word document with the revised version of the manuscript. See Information for Authors for further details.

- The Journal of Physiology funds authors of provisionally accepted papers to use the premium BioRender site to create high resolution schematic figures. Follow this link and enter your details and the manuscript number to create and download figures. Upload these as the figure files for your revised submission. If you choose not to take up this offer, we require figures to be of similar quality and resolution. If you are opting out of this service to authors, state this in the Comments section on the Detailed Information page of the submission form. The link provided should only be used for the purposes of this submission. Authors will be charged for figures created on this premium BioRender account if they are not related to this manuscript submission.

- Please upload separate high-quality figure files via the submission form.

- You must upload original, uncropped western blot/gel images (including controls) if they are not included in the manuscript. This is to confirm that no inappropriate, unethical or misleading image manipulation has occurred. These should be uploaded as 'Supporting information for review process only'. Please label/highlight the original gels so that we can clearly see which sections/lanes have been used in the manuscript figures. For more information, see: https://physoc.onlinelibrary.wiley.com/hub/journal-policies#imagmanip.

- Please ensure that any tables are editable and in Word format, and wherever possible, embedded in the article file itself.

- Please ensure that the Article File you upload is a Word file.

- Papers must comply with the Statistics Policy: https://jp.msubmit.net/cgi-bin/main.plex?form_type=display_requirements#statistics.

In summary:

- If n {less than or equal to} 30, all data points must be plotted in the figure in a way that reveals their range and distribution. A bar graph with data points overlaid, a box and whisker plot or a violin plot (preferably with data points included) are acceptable formats.

- If n > 30, then the entire raw dataset must be made available either as supporting information, or hosted on a not-for-profit repository, e.g. FigShare, with access details provided in the manuscript.

- 'n' clearly defined (e.g. x cells from y slices in z animals) in the Methods. Authors should be mindful of pseudoreplication.

- All relevant 'n' values must be clearly stated in the main text, figures and tables.

- The most appropriate summary statistic (e.g. mean or median and standard deviation) must be used. Standard Error of the Mean (SEM) alone is not permitted.

- Exact p values must be stated. Authors must not use 'greater than' or 'less than'. Exact p values must be stated to three significant figures even when 'no statistical significance' is claimed.

- Please include an Abstract Figure file and an Abstract Figure legend. An appropriate figure legend, which should not exceed 150 words in length, should be included in the main manuscript file. The Abstract Figure is a piece of artwork designed to give readers an immediate understanding of the research and should summarise the main conclusions. If possible, the image should be easily 'readable' from left to right or top to bottom. It should show the physiological relevance of the manuscript so readers can assess the importance and content of its findings. Abstract Figures should not merely recapitulate other figures in the manuscript. Please try to keep the diagram as simple as possible and without superfluous information that may distract from the main conclusion(s). Abstract Figures must be provided by authors no later than the revised manuscript stage and should be uploaded as a separate file during online submission labelled as File Type 'Abstract Figure'. Please also ensure that you include the figure legend in the main article file. All Abstract Figures should be created using BioRender. Authors should use The Journal's premium BioRender account to export high-resolution images. Details on how to use and access the premium account are included as part of this email.

EDITOR COMMENTS

Please also see 'Required Items' above.

Reviewing Editor: Comments to the Author (Required):

Dear Waddell et al.,

Your manuscript has now been evaluated by me and by two external reviewers. There is broad agreement that the model is interesting, the research question is relevant, and that the work is supported by clear expertise. At the same time, both reviewers raised substantial concerns that, in our view, preclude further consideration of the manuscript in its current form. The most important issues relate to the interpretation and robustness of the muscle protein synthesis data, including the possibility that the reported significance may be influenced disproportionately by a potential outlier, as well as concerns regarding the purity of the myofibrillar fraction. In light of these points, the conclusions would also need to more closely reflect the strength and limitations of the data, and the overall tone would need to be moderated accordingly.

REFEREE COMMENTS

Referee #1:

Please attached file.

Referee #2:

General comment:

The study by Waddell et all assessed the impact of vortex wave stimulation "exercise" on muscle physiological outcomes in health older adults. For this study healthy older adults performed a VWS session on two consecutive days. In addition, participants consumed D20 for 7 days to assess muscle protein synthesis rates in two difference muscle fractions. In addition, blood samples were taken to assess inflammatory and bone turnover markers, and myoelectrical activity and muscle oxygenation were assessed during VWS. Where no changes were observed in blood flow and/or anabolic signaling proteins, muscle protein synthesis in the myofibrillar fraction was significantly enhanced by 2 sessions of VWS. Myoelectrical activity increased during VWS, whereas muscle oxygenation decreased. Although the absence of a control group is a limitation, the study employs an elegant design to evaluated (for the first time) the impact of a novel exercise strategy on muscle physiology. The study appears to be well executed by an experienced research group within the field of exercise physiology. The observed significant increase in myofibrillar muscle protein synthesis (MPS) is intriguing. However, its interpretation remains challenging in the absence of a control group performing a more conventional resistance exercise session of comparable duration. Nevertheless, as an initial investigation into this exercise modality, the study provides a valuable foundation for future research in this area. Overall, the manuscript is clearly written. Below, I provide a number of minor comments for the authors to consider.

Specific comments:

In the abstract a rational its stated that VWS may mitigate the decline in skeletal muscle mass during aging and/or disuse. Yet the design of the study does not assess long-term changes in preventing a decline in muscle mass during aging or disuse. It rather explores the acute effects of VWS on muscle physiological outcomes (as stated in the aim). The opening sentence should be more in line with the formulated aim of the study.

Introduction.

In the introduction there is large focus on "sarcopenia", "anabolic resistance" and "disuse" as factors that impact skeletal muscle mass during aging, and that exercise training is an intervention to counteract this. Although this is true, the present study does not include sarcopenic older adults, does not assess anabolic resistance and/or disuse atrophy. Its rather a study that, for the first time, assesses the physiological impact of new exercise training modality (ie. VWS) in healthy older adults. The authors are encouraged to shorten to first two paragraphs of the introduction, and provide more focus towards the VWS per se.

Vortex Wave Stimulation (introduction/methods)

As VWS is a novel exercise strategy, its key that readers understand what the modality specifically entails. Its good that the authors highlight in the introduction that VWS is different compared with the "well-known" whole body vibration" (WBV). The authors provide many details on the on centrifugal forces via an internal rotating disc that are produced by the machine, and extensive details are included on calculations in the supplemental material. But, as a reader, it remains difficult to imagine what the exercise looks like. From image A.3, included in supplemental material, its not possible to get a good idea how it differs from WBV plate, and/or what kind of intensity the exercise projects. I am not sure whether it would be possible, but it would go a long way if, for example, a simple 10 sec video could be included on what VWS exercise looks like in real life. In addition, in the methods its unclear what the exact instruction were that the participants received while standing on the device. Was it just "remain in a standing position"? Were knees/hips fully extended/or slightly in a flexed position? Is a warm-up provided/required? What is the reason for the 2x22.5 min duration of the session?

To get an idea on the exercise intensity it would be of value to known how much heart rate increased during the session? And whether an elevated heart rate was maintained during the entire session? Was RPE assessed at the end of the exercise session? Together this would provide more context towards the novel VMW exercise performed.

Methods.

The primary outcome, MPS, was assessed in the vastus lateralis muscle, which is commonly targeted to evaluate muscle protein synthesis rates. However, it remains unclear to what extent VWS specifically engages this muscle during exercise, given that stability is not the primary function of the vastus lateralis. This raises the question of how representative the measured response is for the exercise modality under investigation.

Visit 6. Please clarify "at a later date". This is too vague. Include for example a range of days.

Diet and physical activity monitoring (section). Please include more details on what/how many specific days food intake diaries were collected, as shown in figure 1. Second, its unclear why food intake was only assessed during the D2O loading phase and not during the baseline and 48h 'VWS phase'? when this is not performed, it cannot be excluded to have a potential impact on the outcome, and should be mentioned as a limitation within the discussion section. Third, please include whether there were any restriction on habitual physical activity level during the baseline and VWS phase of the experiment.

Table 1/text in results: please check appropriateness on the use of decimals for all outcomes. E.g. you do not assess blood pressure in 2 decimals, as such, you should not use them in reporting the mean {plus minus} SD.

Results: its unclear why on protein expression is evaluated in the sarcoplasmic fraction and not the Myofib fraction. Specifically as MPS was only increased in the Myofib and not the SarcoPl fraction.

Due to the explorative nature of the study, the discussion section remains very speculative. Although this is understandable, some suggestions/speculation rather raise more question then potential answers.

In de second paragraph (Pag 27-28) the discussion starts by potentially explaining the increased MyoFib MPS level during VWS, which is suggested to related to myoelectrical activity. 1. This suggestion could be more substantiated if the study would show a positive correlation between increase in myoelectrical activity and increase MPS levels during VWS. 2. The discussion continues to discuss the relationship between EMG and glycogen levels, and that EMG may not be accurate assessment for muscle fiber activation. Still the authors suggest that it may indicate a potential adaptive response, that can be further investigated. Further investigating the accuracy of EMG as a tool to assess muscle fiber activation is beside the point of the current research study. In addition, its unclear why the authors think that measuring muscle glycogen content would be more insightful to determine VWS increased fiber activation. Is VWS that intense that you would expect an drop in glycogen stores?

Paragraph 3. When including integrative MPS rates, this reviewer does not see the added insight of evaluating protein expression at a single time point. As mentioned by the authors the timing of biopsy sampling limits its impact.

Paragraph 4: please include a reference for the statement that sarcoplasmic protein is rapidly replenished.

Paragraph "Lower limb blood flow and oxygen saturation". Here more context is required on the magnitude of changed observes on StO2 and HbR in the present study. What is the magnitude of change is observed in the present study, and how does this relate towards a standard resistance exercise session? Second, the outcome is compared with the Rittweger 2011 and Yamada 2005 WBV studies showing "mild" or "greater" changes. What is considered "mild" and "greater"? and how does this compare with the current study?

In the present study muscle biopsies were collected from the Vastus Lateralis muscle. For understandable reason this muscle is the primary chosen muscle in these kind of studies. However, considering the type of "exercise" exposure, VWS in a standing position will likely target muscle groups that are important for posture, which is not the primary function of the Vastus Lateralis. In the discussion section this not taken into consideration/discussed.

The conclusion paragraph reads more like a summary of the discussion rather than a clearly formulated concluding statement. It would benefit from being more concise and focused on the key takeaway message of the manuscript.

END OF COMMENTS

The manuscript by Waddel et al. is highly relevant for the aging community as it investigates the impact of vortex wave stimulation protocol on physiological responses in aged skeletal muscle. Given the need for practical and efficient intervention tools for maintaining muscle mass in this population, the investigation is an important step forward. However, there are several concerns that need to be resolved.

Major

1. The data on MPS is highly variable, more than usually seen in these types of investigations, which is concerning. This in turn may have influenced the statistical outcome, and the subsequent conclusions. First, given the untrained nature of the population, I would expect an increase in both fractions, if any. Yet there was only a statistical increase in the myofibrillar fraction, even though there was a numerical increase in sarcoplasmic fraction. Looking at the individual data, one participant in particular appears to drive the majority of increase in iMyoPS. Have the authors examined the statistical data without this high-responder individual?
2. The high variability has received no attention at all in the manuscript which is concerning. At first glance, the study population appears to be of good size. However, the power calculations are based on outcomes from a study in which resistance exercise was used as an intervention. Arguably, RE would be much more potent in increasing MPS compared to VWS. Yet, in this study, the increase is much larger (~30%) compared to ~9% in McKendry et al 2019. This further suggests that the increase seen here more likely reflects a type I error (false positive) due to high variation and lack of statistical power, rather than a true increase. I recommend the authors perform post-hoc power calculations without the single high-responder to determine the actual power of the study.
3. Given the continuous nature of the VWS protocol, it more resembles endurance exercise rather than resistance exercise. It is therefore more plausible that the significant increase, assuming it is true, is more representative of increased mitochondrial protein synthesis. While the authors partly argue that the higher numeric values of MPS in the cytosolic fraction are due in part to mitochondria in that fraction, I believe this may be incorrect. Based on the methods description, I suspect the myofibrillar fraction actually contains a large number of mitochondria. Most common protocols for separation of myofibrillar, mitochondrial and cytosolic fractions involve differential centrifugation steps. Here, muscle was homogenized in buffer without a detergent, leaving mitochondria intact and interconnected which together makes them heavy in comparison to non-compartmentalized cytosolic proteins. Under these conditions, the myofibrillar fraction would be centrifuged around 1000 g to pellet only myofibrillar proteins with minimal mitochondrial contamination. The resulting supernatant would then be centrifuged at >10 000 g to

pellet mitochondria. That resulting supernatant would then be considered the cytosolic fraction. In the present study, samples were centrifuged at 11 000 g after homogenization, i.e. at a centrifugal speed where intact mitochondria are pelleted together with the myofibrillar proteins. This potential confounding factor needs to be resolved for a correct conclusion. If this is indeed the case, the VWS protocol may actually be more suitable for endurance-type adaptations, which in themselves are highly beneficial for older adults. If so, the manuscript should be re-focused towards endurance-type exercise, rather than muscle growth. The authors should perform purity checks with mitochondrial and myofibrillar markers side-by-side in both fractions. This can easily be done on just a few samples and would be a great addition to the manuscript.

4. Lastly, the high variability could potentially be due to the homogenization protocol employed (in addition to the point above). The buffer used for homogenization lacks protease inhibitors, which is very concerning. Homogenates remain without these inhibitors for at least 25 minutes after homogenization (10 min shaking + 15 min centrifugation) and are only added to the cytosolic fraction after centrifugation. Please explain the reasoning behind this protocol and I would recommend to address this in the limitations section.

Minor

1. Data on muscle glycogen would significantly strengthen the manuscript, as would measurements of blood lactate. Providing these data would allow for a deeper physiological examination of the effects on VWS. Glycogen can easily be measured on miniscule amounts of muscle and lactate levels can remain elevated more than an hour after an exercise session so it would be interesting to measure lactate before and 30 min post VWS.

Editor's comments:

Your manuscript has now been evaluated by me and by two external reviewers. There is broad agreement that the model is interesting, the research question is relevant, and that the work is supported by clear expertise. At the same time, both reviewers raised substantial concerns that, in our view, preclude further consideration of the manuscript in its current form. The most important issues relate to the interpretation and robustness of the muscle protein synthesis data, including the possibility that the reported significance may be influenced disproportionately by a potential outlier, as well as concerns regarding the purity of the myofibrillar fraction. In light of these points, the conclusions would also need to more closely reflect the strength and limitations of the data, and the overall tone would need to be moderated accordingly.

We would like to thank the editor and reviewers for taking the time to review our manuscript and providing encouraging comments about the potential of the article. In response to your queries, we have addressed the concern over the potential influence of a high responding outlier by performing sensitivity analyses. Although the magnitude of effect was predictably reduced, statistical significance remained. We also provide additional data concerning the purity of our myofibrillar fraction. We demonstrate the presence of mitochondrial complexes in our sarcoplasmic fraction, as well as greater citrate synthase activity in the sarcoplasmic vs myofibrillar fractions. Taken together, we feel these data suggest the contributions of mitochondria are limited and unlikely to significantly influence the observed increase in iMyoPS. Finally, we have amended the overall tone of the manuscript, with greater emphasis on the acute, exploratory nature of the study, and more transparency on the limitations. We hope our individual responses to each of the reviewers concerns below are sufficient.

Reviewer 1 comments:

The manuscript by Waddell et al. is highly relevant for the aging community as it investigates the impact of vortex wave stimulation protocol on physiological responses in aged skeletal muscle. Given the need for practical and efficient intervention tools for maintaining muscle mass in this population, the investigation is an important step forward. However, there are several concerns that need to be resolved.

Thank you for your encouraging comments on our manuscript. We hope we have provided sufficient further information below in response to your queries.

The data on MPS is highly variable, more than usually seen in these types of investigations, which is concerning. This in turn may have influenced the statistical outcome, and the subsequent conclusions. First, given the untrained nature of the population, I would expect an increase in both fractions, if any. Yet there was only a

statistical increase in the myofibrillar fraction, even though there was a numerical increase in sarcoplasmic fraction. Looking at the individual data, one participant in particular appears to drive the majority of increase in iMyoPS. Have the authors examined the statistical data without this high-responder individual?

After statistically identifying the high responder as an outlier, we removed them from the dataset in a sensitivity analysis. The statistical significance remained, albeit reduced in magnitude. We have now updated the results to highlight this, with updated mean difference, confidence intervals and effect size. A similar pattern was observed with the EMG data, with one high responder identified as an outlier. Likewise, after removing their data the statistical significance remained, which we have similarly highlighted in the results. We have now also updated our statistical analysis in the methods to reflect these additions.

The high variability has received no attention at all in the manuscript which is concerning. At first glance, the study population appears to be of good size. However, the power calculations are based on outcomes from a study in which resistance exercise was used as an intervention. Arguably, RE would be much more potent in increasing MPS compared to VWS. Yet, in this study, the increase is much larger (~30%) compared to ~9% in McKendry et al 2019. This further suggests that the increase seen here more likely reflects a type I error (false positive) due to high variation and lack of statistical power, rather than a true increase. I recommend the authors perform post-hoc power calculations without the single high-responder to determine the actual power of the study.

We acknowledge the reviewers concern regarding the variability of the MPS data. While the observed increase in iMyoPS is larger than what might be expected following resistance exercise, the variability is consistent with what has been previously reported by our group using the same methodology (Marshall et al., 2023). Furthermore, although there is variation in individual values, the response is generally consistent in direction, regardless of the high-responding outlier. We acknowledge the confidence in the observed effect is limited by the sample size and variability and have now addressed this in the manuscript discussion. However, we do not believe the variability alone is evidence that the observed significant increase in iMyoPS represents a type I error.

The observed post hoc power was 0.67 without the high responding outlier. However, we note that statistical power and the type I error rate are distinct concepts. The Type I error rate is determined by the pre-specified α level, while power reflects the probability of detecting a true effect. As such, a post hoc power analysis does not directly inform whether the significant increase in iMyoPS represents a false-positive. We therefore focus our interpretation on the observed effect, variability in the data, and the consistency of the response following VWS.

Given the continuous nature of the VWS protocol, it more resembles endurance exercise rather than resistance exercise. It is therefore more plausible that the significant increase, assuming it is true, is more representative of increased mitochondrial protein synthesis. While the authors partly argue that the higher numeric values of MPS in the cytosolic fraction are due in part to mitochondria in that fraction, I believe this may be incorrect. Based on the methods description, I suspect the myofibrillar fraction actually contains a large number of mitochondria. Most common protocols for separation of myofibrillar, mitochondrial and cytosolic fractions involve differential centrifugation steps. Here, muscle was homogenized in buffer without a detergent, leaving mitochondria intact and interconnected which together makes them heavy in comparison to non-compartmentalized cytosolic proteins. Under these conditions, the myofibrillar fraction would be centrifuged around 1000 g to pellet only myofibrillar proteins with minimal mitochondrial contamination. The resulting supernatant would then be centrifuged at >10 000 g to pellet mitochondria. That resulting supernatant would then be considered the cytosolic fraction. In the present study, samples were centrifuged at 11 000 g after homogenization, i.e. at a centrifugal speed where intact mitochondria are pelleted together with the myofibrillar proteins. This potential confounding factor needs to be resolved for a correct conclusion. If this is indeed the case, the VWS protocol may actually be more suitable for endurance-type adaptations, which in themselves are highly beneficial for older adults. If so, the manuscript should be re-focused towards endurance-type exercise, rather than muscle growth. The authors should perform purity checks with mitochondrial and myofibrillar markers side-by-side in both fractions. This can easily be done on just a few samples and would be a great addition to the manuscript.

We have attempted to address the reviewer's query regarding the potential influence of mitochondrial proteins within the myofibrillar fraction using remaining available tissue and consumables. Citrate synthase (CS) activity was detectable in the sarcoplasmic fraction (0.041 $\mu mol \cdot min^{-1} \cdot mg^{-1}$), whereas activity in the myofibrillar fraction was negligible (−0.016 $\mu mol \cdot min^{-1} \cdot mg^{-1}$). In addition, immunoblotting of the sarcoplasmic fraction using a Total OXPHOS antibody cocktail confirmed the presence of mitochondrial proteins. Complexes V, III and II were readily detected, whereas Complexes I and IV were comparatively faint, consistent with observations from the antibody manufacturer that sample heating can reduce detection of these proteins. Due to sample availability, the isolation protocol and low protein content, we were not able to carry out a similar blot on the myofibrillar fraction.

We acknowledge that the lack of detectable CS activity in the myofibrillar fraction may reflect loss of enzyme integrity during alkaline hydrolysis with NaOH to solubilise the myofibrillar proteins. Nevertheless, the combination of measurable CS activity and detectable OXPHOS proteins in the sarcoplasmic fraction, together with negligible CS activity in the myofibrillar fraction, suggests that mitochondrial contributions to the myofibrillar protein pool are limited and are therefore unlikely to explain the observed increase in iMyoPS and unchanged iSarcPS. We have provided the additional methods and results in the appendix and referenced this work in the discussion.

Lastly, the high variability could potentially be due to the homogenization protocol employed (in addition to the point above). The buffer used for homogenization lacks protease inhibitors, which is very concerning. Homogenates remain without these inhibitors for at least 25 minutes after homogenization (10 min shaking + 15 min centrifugation) and are only added to the cytosolic fraction after centrifugation. Please explain the reasoning behind this protocol and I would recommend to address this in the limitations section.

We omitted protease inhibitors from the homogenisation buffer to avoid contamination of downstream amino acid quantification by mass spectrometry in the sarcoplasmic fraction. The standard protocol is to wash the myofibrillar pellet with ddH_2O and buffer prior to elution to prevent contamination, however the sarcoplasmic fraction cannot be washed in this way. We then subsequently added protease inhibitors to a smaller portion of sarcoplasmic fraction set aside for immunoblotting to preserve protein integrity. To mitigate proteolysis, samples were processed rapidly on ice, with ice-cold homogenisation buffer and fractionated as quickly as possible. We have now addressed this aspect of the protocol in the limitations, however we point out that all samples were treated identically, and so do not think it is likely to explain the variability in the dataset.

Data on muscle glycogen would significantly strengthen the manuscript, as would measurements of blood lactate. Providing these data would allow for a deeper physiological examination of the effects on VWS. Glycogen can easily be measured on miniscule amounts of muscle and lactate levels can remain elevated more than an hour after an exercise session so it would be interesting to measure lactate before and 30 min post VWS.

We now provide additional data on blood lactate and muscle glycogen measurements as suggested. We were also able to collect blood glucose measures at the same time. We have integrated the findings into the results and discussion. We have slightly reordered the discussion based on the additional findings.

Reviewer 2 comments:

The study by Waddell et all assessed the impact of vortex wave stimulation "exercise" on muscle physiological outcomes in health older adults. For this study healthy older adults performed a VWS session on two consecutive days. In addition, participants consumed D20 for 7 days to assess muscle protein synthesis rates in two difference muscle fractions. In addition, blood samples were taken to assess inflammatory and bone turnover markers, and myoelectrical activity and muscle oxygenation were assessed during VWS. Where no changes

were observed in blood flow and/or anabolic signaling proteins, muscle protein synthesis in the myofibrillar fraction was significantly enhanced by 2 sessions of VWS. Myoelectrical activity increased during VWS, whereas muscle oxygenation decreased. Although the absence of a control group is a limitation, the study employs an elegant design to evaluated (for the first time) the impact of a novel exercise strategy on muscle physiology. The study appears to be well executed by an experienced research group within the field of exercise physiology. The observed significant increase in myofibrillar muscle protein synthesis (MPS) is intriguing. However, its interpretation remains challenging in the absence of a control group performing a more conventional resistance exercise session of comparable duration. Nevertheless, as an initial investigation into this exercise modality, the study provides a valuable foundation for future research in this area. Overall, the manuscript is clearly written. Below, I provide a number of minor comments for the authors to consider.

Thank you for your encouraging comments on our manuscript. We hope we have provided sufficient responses and/or additional information following your suggestions.

In the abstract a rational its stated that VWS may mitigate the decline in skeletal muscle mass during aging and/or disuse. Yet the design of the study does not assess long-term changes in preventing a decline in muscle mass during aging or disuse. It rather explores the acute effects of VWS on muscle physiological outcomes (as stated in the aim). The opening sentence should be more in line with the formulated aim of the study.

As suggested, we have now amended the ending of the abstract to more accurately reflect the focus of the manuscript. We have removed the statements suggesting any potential mitigation of skeletal muscle mass during ageing and/or disuse and have highlighted this is the first study to demonstrate the acute physiological responses to VWS.

In the introduction there is large focus on "sarcopenia", "anabolic resistance" and "disuse" as factors that impact skeletal muscle mass during aging, and that exercise training is an intervention to counteract this. Although this is true, the present study does not include sarcopenic older adults, does not assess anabolic resistance and/or disuse atrophy. Its rather a study that, for the first time, assesses the physiological impact of new exercise training modality (ie. VWS) in healthy older adults. The authors are encouraged to shorten to first two paragraphs of the introduction, and provide more focus towards the VWS per se.

We have now reduced the length of the first two paragraphs, with less emphasis on disuse, obesity, sarcopenia and anabolic resistance. However, we feel that the rationale for examining the effects of VWS in older adults specifically in the present study warrants retaining some mention of sarcopenia/osteoporosis, to justify the population studied and the outcomes measured. We have also added a statement at the end of the second to last

paragraph to add to the rationale for studying the acute effects of VWS (i.e. to feed into a rationale for more long-term study).

As VWS is a novel exercise strategy, its key that readers understand what the modality specifically entails. Its good that the authors highlight in the introduction that VWS is different compared with the "well-known" whole body vibration" (WBV). The authors provide many details on the on centrifugal forces via an internal rotating disc that are produced by the machine, and extensive details are included on calculations in the supplemental material. But, as a reader, it remains difficult to imagine what the exercise looks like. From image A.3, included in supplemental material, its not possible to get a good idea how it differs from WBV plate, and/or what kind of intensity the exercise projects. I am not sure whether it would be possible, but it would go a long way if, for example, a simple 10 sec video could be included on what VWS exercise looks like in real life. In addition, in the methods its unclear what the exact instruction were that the participants received while standing on the device. Was it just "remain in a standing position"? Were knees/hips fully extended/or slightly in a flexed position? Is a warm-up provided/required? What is the reason for the 2x22.5 min duration of the session?

We have also now provided more detail in the fourth paragraph surrounding VWS, to provide more context for what the stimulus may look like. In the methods section, we have provided more detail on how participants were instructed during the VWS sessions. The sessions were based on what is typically undertaken within private clinics providing VWS to their patients. There is no warmup necessary, patients typically stand in a position that is ‘comfortable’, which lasts for 45 minutes, with short rest periods if needed. The duration was thus 22.5 mins to reach a total of 45 mins with a short rest in-between.

To get an idea on the exercise intensity it would be of value to known how much heart rate increased during the session? And whether an elevated heart rate was maintained during the entire session? Was RPE assessed at the end of the exercise session? Together this would provide more context towards the novel VMW exercise performed.

Unfortunately, we did not record heart rate during the VWS sessions, nor did we have a rating of perceived exertion assessment. The only qualitative measure we recorded was that of the visual analogue scale of muscle soreness before and after each session. We have added to the lower limb blood flow section that recording heart rate would have facilitated a more comprehensive haemodynamic assessment and updated the limitations section that without RPE and heart rate we were not able to quantify the ‘intensity’.

The primary outcome, MPS, was assessed in the vastus lateralis muscle, which is commonly targeted to evaluate muscle protein synthesis rates. However, it remains unclear to what extent VWS specifically engages this muscle during exercise, given that stability is not the

primary function of the vastus lateralis. This raises the question of how representative the measured response is for the exercise modality under investigation.

While we agree the stationary nature of VWS may mean the vastus lateralis is not the most reflective muscle to measure, as you point out it is frequently used to measure muscle protein synthesis. This allows us to pair muscle protein synthesis directly with other outcomes at the same site. Indeed, we do see consistent elevations in EMG activity at the vastus lateralis during VWS which may indicate involvement, although we also discuss the limitations of this measure at length. We also observe comparable rates of deoxygenation between the vastus lateralis and gastrocnemius, which plays a greater role in stability – we have added this point to the limitations section, in addition to acknowledging how other muscle groups were neglected in our assessments. We also note that the thigh muscles are more susceptible to age-related atrophy (10.1016/j.exger.2023.112159), further emphasising the need to explore the effects of different interventions on these muscles.

Visit 6. Please clarify "at a later date". This is too vague. Include for example a range of days.

We have updated the methods here to provide the range of days between visits 5 and 6.

Diet and physical activity monitoring (section). Please include more details on what/how many specific days food intake diaries were collected, as shown in figure 1. Second, its unclear why food intake was only assessed during the D2O loading phase and not during the baseline and 48h 'VWS phase'? when this is not performed, it cannot be excluded to have a potential impact on the outcome, and should be mentioned as a limitation within the discussion section. Third, please include whether there were any restriction on habitual physical activity level during the baseline and VWS phase of the experiment.

We have added more detail surrounding the food diaries. We used a standardised weighed food diary that participants use to record their food intake over three days. This is to provide a representative account of their habitual intake. Participants are asked to continue with their typical intake for the remainder of the study. This reduces burden on participant compared to asking them to weigh and record intake across the entire study duration. However, we do acknowledge that this means we cannot be certain food intake did not change between the different phases of the study and have updated the limitations sections to reflect this. We have added to the methods section that in addition to avoiding strenuous exercise, participants were asked to continue with their habitual physical activity. Participants wore accelerometers throughout, and we mention in the first paragraph of the results that step count was similar across the different phases.

Table 1/text in results: please check appropriateness on the use of decimals for all outcomes. E.g. you do not assess blood pressure in 2 decimals, as such, you should not use them in reporting the mean {plus minus} SD.

As suggested, we have amended the table so that decimal places are utilised appropriately.

Results: its unclear why on protein expression is evaluated in the sarcoplasmic fraction and not the Myofib fraction. Specifically as MPS was only increased in the Myofib and not the SarcoPl fraction.

The rationale for exploring protein expression in the sarcoplasmic fraction was to examine whether there were any changes in the signalling pathway that mediates rates of myofibrillar protein synthesis, rather than exploring individual myofibrillar proteins. By investigating up- and downstream targets of the mTOR signalling cascade, which are located in the sarcoplasm, we sought to potentially help explain the increase in iMyoPS. We also investigated expression of Focal Adhesion Kinase to explore potential detection of mechanical tension. As we mention later in the discussion, the biopsy timings required for assessing iMyoPS and iSarcPS limit a more comprehensive examination of changes in protein expression, which are typically much shorter lived. This assessment was therefore quite exploratory. We have added this rationale to the discussion and acknowledge that exploring the expression of individual myofibrillar proteins would have provided additional insight.

In de second paragraph (Pag 27-28) the discussion starts by potentially explaining the increased MyoFib MPS level during VWS, which is suggested to related to myoelectrical activity. 1. This suggestion could be more substantiated if the study would show a positive correlation between increase in myoelectrical activity and increase MPS levels during VWS. 2. The discussion continues to discuss the relationship between EMG and glycogen levels, and that EMG may not be accurate assessment for muscle fiber activation. Still the authors suggest that it may indicate a potential adaptive response, that can be further investigated. Further investigating the accuracy of EMG as a tool to assess muscle fiber activation is beside the point of the current research study. In addition, its unclear why the authors think that measuring muscle glycogen content would be more insightful to determine VWS increased fiber activation. Is VWS that intense that you would expect an drop in glycogen stores?

We have performed a correlation analysis between the changes in EMG and iMyoPS as suggested, which was not significant. We have incorporated these results into the discussion to counteract suggesting the increase in myoelectrical activity is directly indictive of neuromuscular activation that is driving the increase in iMyoPS. In relation to your second point, we did not mean to suggest the accuracy of EMG as a tool to assess fibre activation could be investigated further. We were suggesting that as surface EMG is a simple tool, it may be used in studies like ours, and if there is some indication of changes in the signal, this could

warrant further investigation of the stimulus being explored with more invasive or complex techniques. We have amended the text to make this point clearer. We initially referred to the possibility of exploring changes in muscle glycogen content as previous work suggests it is a more accurate indicator of fibre activation compared to surface EMG. However, we have subsequently measured muscle glycogen in our samples following a comment from the other reviewer and have integrated these results into the manuscript.

Paragraph 3. When including integrative MPS rates, this reviewer does not see the added insight of evaluating protein expression at a single time point. As mentioned by the authors the timing of biopsy sampling limits its impact.

As we mentioned in relation to one of your previous points, we acknowledge the timings of the biopsies, which were chosen to evaluate integrated rates of protein synthesis, limit a more comprehensive assessment of changes in protein expression. To clarify, we did not assess protein expression at a single time point, we chose to explore potential changes in protein expression between visit 3 (biopsy prior to the first session of VWS) and visit 5 (biopsy after 2 sessions of VWS had been completed), to explore if there were any sustained elevations in protein expression/phosphorylation in the signalling pathway that regulates muscle protein synthesis.

Paragraph 4: please include a reference for the statement that sarcoplasmic protein is rapidly replenished.

As suggested, we have updated the statement and added a reference.

Paragraph "Lower limb blood flow and oxygen saturation". Here more context is required on the magnitude of changed observes on StO2 and HbR in the present study. What is the magnitude of change is observed in the present study, and how does this relate towards a standard resistance exercise session? Second, the outcome is compared with the Rittweger 2011 and Yamada 2005 WBV studies showing "mild" or "greater" changes. What is considered "mild" and "greater"? and how does this compare with the current study?

We have added some of our data to this section in the discussion to highlight the magnitude of change in StO_2 and Hbr. We have also added how this compares to studies using WBV, removing the words 'mild' and 'greater' (we have replaced the reference of Rittweger et al 2011, as on reflection the mention of oxygen uptake during WBV in Rittweger et al 2011 is less relevant to our data as it is not something we explored). We have now also added in some data on the effects of exercise on StO_2 and Hbr to contextualise the magnitude of effect we observed.

In the present study muscle biopsies were collected from the Vastus Lateralis muscle. For understandable reason this muscle is the primary chosen muscle in these kind of studies. However, considering the type of "exercise" exposure, VWS in a standing position will likely target muscle groups that are important for posture, which is not the primary function of the Vastus Lateralis. In the discussion section this not taken into consideration/discussed.

As you have stated, we focused our assessments on the Vastus lateralis as this is where the majority of this type of research tends to focus and is what has typically been done in our laboratory. As mentioned in response to an earlier comment, we point out that we observed comparable muscle deoxygenation at the gastrocnemius and have added to the limitations that other muscle groups responsible for maintaining posture were not assessed, and given the nature of VWS requires participants to remain standing for up to 45 minutes, this may have provided greater insight.

The conclusion paragraph reads more like a summary of the discussion rather than a clearly formulated concluding statement. It would benefit from being more concise and focused on the key takeaway message of the manuscript.

On reflection we agree with your comment about the conclusion. We have now amended the section as suggested.

Dear Dr Breen,

Re: JP-RP-2026-291437R1 "**Musculoskeletal and physiological responses to vortex wave stimulation in older adults**" by Alexander Waddell, Marie Korzepa, Archie E Belfield, Ben Fry, Rickson C. Mesquita, David John Smith, Jonathan I Quinlan, and Leigh Breen

Thank you for submitting your manuscript to The Journal of Physiology. It has been assessed by a Reviewing Editor and by 2 expert referees and we are pleased to tell you that it is acceptable for publication following satisfactory revision.

Please share this decision with your co-authors as soon as possible.

The referee reports are copied at the end of this email.

Please address all the points raised and incorporate all requested revisions or explain in your Response to Referees why a change has not been made. We hope you will find the comments helpful and that you will be able to return your revised manuscript within 4 weeks. If you require longer than this, please contact journal staff: jp@physoc.org.

Your revised manuscript should be submitted online using the link in your Author Tasks Link Not Available. This link is accessible via your account as Corresponding Author; it is not available to your co-authors. If this presents a problem, please contact journal staff (jp@physoc.org). Image files from the previous version are retained on the system. Please ensure you replace or remove any files that are being revised.

If you do not wish to submit a revised version of your manuscript, you must inform our journal staff (jp@physoc.org) or reply to this email to request withdrawal. Please note that a manuscript must be formally withdrawn from the peer review process at one journal before it may be submitted to another journal.

TRANSPARENT PEER REVIEW POLICY: To improve the transparency of its peer review process The Journal of Physiology publishes online, as supporting information, the peer review history of all articles accepted for publication. Readers will have access to decision letters, including Editors' comments and referee reports, for each version of the manuscript, as well as any author responses to peer review comments. Referees can decide whether or not they wish to be named on the peer review history document.

ABSTRACT FIGURES: Authors are expected to use The Journal's premium BioRender account to create/redraw their Abstract Figures. Information on how to access this account is here: https://physoc.onlinelibrary.wiley.com/journal/14697793/biorender-access.

This will enable Authors to create and download high-resolution figures. If authors have used the free BioRender service, they can use the instructions provided in the link above to download a high-resolution version suitable for publication.

The link provided should only be used for the purposes of this submission. Authors will be charged for figures created on this account if they are not related to this manuscript submission.

LANGUAGE EDITING AND SUPPORT FOR PUBLICATION: If you would like help with English language editing, or other article preparation support, Wiley Editing Services offers expert help, including English Language Editing, as well as translation, manuscript formatting, and figure formatting at www.wileyauthors.com/eeo/preparation. You can also find resources for Preparing Your Article for general guidance about writing and preparing your manuscript at www.wileyauthors.com/eeo/prepresources.

RIGOUR AND REPRODUCIBILITY: We recommend authors consult The Journal's Rigour and Reproducibility requirements to ensure that their manuscript is suitable for publication: https://physoc.onlinelibrary.wiley.com/pb-assets/hub-assets/physoc/documents/TJP-Rigour-and-Reproducibility-Requirements.pdf

REVISION CHECKLIST:

Check that your Methods section conforms to journal policy: https://jp.msubmit.net/cgi-bin/main.plex?form_type=display_requirements#methods

Check that data presented conforms to the statistics policy: https://jp.msubmit.net/cgi-bin/main.plex?form_type=display_requirements#statistics - PLEASE CHECK Figure A5 - mentions SEM/error bars. SD should be used in place of SEM.

Upload a full Response to Referees file. To create your 'Response to Referees' copy all the reports, including any comments from the Senior and Reviewing Editors, into a Microsoft Word, or similar, file and respond to each point, using font or background colour to distinguish comments and responses and upload as the required file type.

Please upload two versions of your manuscript text: one with all relevant changes highlighted and one clean version with no

changes tracked. The manuscript file should include all tables and figure legends, but each figure/graph should be uploaded as separate, high-resolution files. The journal is now integrated with Wiley's Image Checking service. For further details, see: https://www.wiley.com/en-us/network/publishing/research-publishing/trending-stories/upholding-image-integrity-wileys-image-screening-service

You may also upload:

- 'Potential Cover Art' for consideration as the issue's cover image
- Appropriate Supporting Information (Video, audio or data set: see https://jp.msubmit.net/cgi-bin/main.plex?form_type=display_requirements#supp).

We look forward to receiving your revised submission.

If you have any queries, please reply to this email and we will be pleased to advise.

Yours sincerely,

Bettina Mittendorfer
Senior Editor
The Journal of Physiology

EDITOR COMMENTS

Reviewing Editor:

Comments to the Author (Required):
Dear Waddell et al.,

Your manuscript has now been re-evaluated by the original reviewers and by me. We appreciate the careful efforts made to address the concerns raised during the first round of review, including the additional data provided and the clarifications regarding the muscle protein synthesis measurements. The revision has improved the manuscript substantially. However, one final important point remains, as raised by Reviewer 1. Please address this directly in both the manuscript and the response letter.

REFEREE COMMENTS

Referee #1:

I commend the authors for their efforts to address my concerns, well done!

However, I have one more minor comment that needs addressing. Please see the attached file.

Referee #2:

The authors appropriately addressed the comments raised by the reviewers, and revised the manuscript accordingly. I have no further comments.

END OF COMMENTS

I commend the authors for their efforts to address my concerns, well done!

However, I have one more minor comment that needs addressing.

In their rebuttal the authors state the following:

"Nevertheless, the combination of measurable CS activity and detectable OXPHOS proteins in the sarcoplasmic fraction, together with negligible CS activity in the myofibrillar fraction, suggests that mitochondrial contributions to the myofibrillar protein pool are limited and are therefore unlikely to explain the observed increase in iMyoPS and unchanged iSarcPS."

I do not agree with this statement and it needs to be changed. The lack of CS activity in the myofibrillar fraction, very likely due to the solubilization with NaOH, needs to be acknowledged in the manuscript as a limitation, as well as the lack of mitochondrial complex proteins measured in the myofibrillar fraction. As it stands, the only conclusion that can be drawn is that mitochondria and CS activity is present in the sarcoplasmic fraction. Nothing can be said about the myofibrillar fraction due the reasons mentioned above. This is a very important distinction.

Editor

Your manuscript has now been re-evaluated by the original reviewers and by me. We appreciate the careful efforts made to address the concerns raised during the first round of review, including the additional data provided and the clarifications regarding the muscle protein synthesis measurements. The revision has improved the manuscript substantially. However, one final important point remains, as raised by Reviewer 1. Please address this directly in both the manuscript and the response letter

We would like to thank both the editor and the two reviewers for their positive and constructive feedback throughout the review process. We have now updated the manuscript as reviewer 1 has suggested.

Reviewer 1

I commend the authors for their efforts to address my concerns, well done!

However, I have one more minor comment that needs addressing.

In their rebuttal the authors state the following:

"Nevertheless, the combination of measurable CS activity and detectable OXPHOS proteins in the sarcoplasmic fraction, together with negligible CS activity in the myofibrillar fraction, suggests that mitochondrial contributions to the myofibrillar protein pool are limited and are therefore unlikely to explain the observed increase in iMyoPS and unchanged iSarcPS."

I do not agree with this statement and it needs to be changed. The lack of CS activity in the myofibrillar fraction, very likely due to the solubilization with NaOH, needs to be acknowledged in the manuscript as a limitation, as well as the lack of mitochondrial complex proteins measured in the myofibrillar fraction. As it stands, the only conclusion that can be drawn is that mitochondria and CS activity is present in the sarcoplasmic fraction. Nothing can be said about the myofibrillar fraction due the reasons mentioned above. This is a very important distinction.

We would again like to thank the reviewer for their positive and constructive comments throughout the review process. As suggested, we have added to the manuscript in Appendix B, highlighting the likely contribution of the solubilisation in NaOH to the CS activity data. We also highlight the lack of an accompanying OXPHOS blot on the myofibrillar fraction. We have added a final sentence to this section, concluding that we demonstrate the presence of mitochondrial complex proteins and CS activity in the sarcoplasmic fraction, with no mention of the myofibrillar fraction.

Reviewer 2

The authors appropriately addressed the comments raised by the reviewers, and revised the manuscript accordingly. I have no further comments.

We would like to thank the reviewer for the constructive feedback throughout the review process.

Dear Professor Breen,

Re: JP-RP-2026-291437R2 "**Musculoskeletal and physiological responses to vortex wave stimulation in older adults**" by Alexander Waddell, Marie Korzepa, Archie E Belfield, Ben Fry, Rickson C. Mesquita, David John Smith, Jonathan I Quinlan, and Leigh Breen

We are pleased to tell you that your paper has been accepted for publication in The Journal of Physiology.

The last Word (or similar) version of the manuscript provided will be used by the Production Editor to prepare your proof. When this is ready you will receive an email containing a link to Wiley's Online Proofing System. The proof should be thoroughly checked and corrected as promptly as possible.

Authors should note that it is too late at this point to offer corrections prior to proofreading. Major corrections at proof stage, such as changes to figures, will be referred to the Editors for approval before they can be incorporated. Only minor changes, such as to style and consistency, should be made at proof stage. Changes that need to be made after proof stage will usually require a formal correction notice.

All queries at proof stage should be sent to: TJP@wiley.com.

Following copyediting and typesetting the accepted version will be published online.

Yours sincerely,

Bettina Mittendorfer
Senior Editor
The Journal of Physiology

IMPORTANT POINTS TO NOTE FOLLOWING ACCEPTANCE OF YOUR PAPER:

• IMPORTANT NOTICE ABOUT OPEN ACCESS: To assist authors whose funding agencies mandate immediate public access to published research findings, The Journal of Physiology allows authors to pay an Open Access (OA) fee to have their papers made freely available immediately on publication.

The Corresponding Author will receive an email from Wiley with details on how to register or log in to Wiley Authors where you will be able to place an order.

• You can check if your funder or institution has a Wiley Open Access Account here:
https://authors.wiley.com/author-resources/Journal-Authors/open-access/author-compliance-tool.html

MEMBER DISCOUNT: Corresponding authors who are Members of the Physiological Society are eligible for a 20% discount on the cost of the Article Processing Charge (APC). To claim this discount, the Corresponding Author will need to log in to the Physiological Society Membership portal and retrieve the discount code (https://www.physoc.org/journals-and-media/journals/journal-discount-for-members/). This code should then be quoted in the billing section of Wiley Authors when selecting the open access option.

• TRANSPARENT PEER REVIEW POLICY: To improve the transparency of its peer review process, The Journal of Physiology publishes online as supporting information the peer review history of all articles accepted for publication. Readers will have access to decision letters, including Editors' comments and referee reports, for each version of the manuscript as well as any author responses to peer review comments. Referees can decide whether or not they wish to be named on the peer review history document.

• You can help your research get the attention it deserves! Check out Wiley's free Promotion Guide for best-practice recommendations for promoting your work at: www.wileyauthors.com/eeo/guide. You can learn more about Wiley Editing Services which offers professional video, design, and writing services to create shareable video abstracts, infographics, conference posters, lay summaries, and research news stories for your research at: www.wileyauthors.com/eeo/promotion.

• If you would like to receive our 'Research Roundup', a monthly newsletter highlighting the cutting-edge research published in The Physiological Society's family of journals (The Journal of Physiology, Experimental Physiology, Physiological Reports, The Journal of Nutritional Physiology and The Journal of Precision Medicine: Health and Disease), please click this link, fill in your name and email address and select 'Research Roundup':
https://www.physoc.org/journals-and-media/membernews

EDITOR COMMENTS

Reviewing Editor:

Comments to the Author (Required):
The final comment has been addressed.
